# A single‐molecule prodrug synergistically suppresses MYC‐amplified osteosarcoma through sequential nitric oxide release and photodynamic therapy

**DOI:** 10.1002/smo2.70084

**Published:** 2026-08-03

**Authors:** Shuxin Peng, Jiangpeng Wu, Shasha Wang, Ying Gao, Xuran Guo, Mengpan Li, Youjin Wu, Yun Wang, Zhengdong Cai, Yingqi Hua, Peng Wei, Yinghua Gao, Tao Yi

**Affiliations:** ^1^ State Key Laboratory of Advanced Fiber Materials College of Chemistry and Chemical Engineering Donghua University Shanghai China; ^2^ Department of Orthopedic Oncology Shanghai Bone Tumor Institute Shanghai General Hospital Shanghai Jiao Tong University School of Medicine Shanghai China; ^3^ Department of Orthopaedics Jinshan Hospital Fudan University Shanghai China

**Keywords:** MYC, nitric oxide, osteosarcoma, photodynamic therapy, prodrug

## Abstract

MYC‐amplified osteosarcoma, a poor‐prognosis molecular subtype, presents formidable therapeutic challenges due to its aggressive phenotype, chemoresistance, and the “undruggable” oncogenic driver MYC. A single‐molecule prodrug, DHU‐NO3, was developed to achieve precise synergy between photodynamic therapy (PDT) and nitric oxide (NO) gas therapy for the treatment of MYC‐amplified osteosarcoma. This prodrug covalently links the clinically approved photosensitizer methylene blue (MB) to an NO donor and undergoes a sequential activation cascade: reactive oxygen species‐triggered MB release, 405 nm light‐controlled NO generation, and PDT initiation under 650 nm laser irradiation. In 143B osteosarcoma cells, this temporally coordinated regimen efficiently induces apoptosis and potently suppresses the MYC signaling network. In the 143B osteosarcoma subcutaneous xenograft model, DHU‐NO3‐mediated sequential phototherapy demonstrated robust tumor growth inhibition with favorable biosafety. This work establishes a spatiotemporally programmable prodrug platform and provides a potent strategy to combat MYC‐amplified osteosarcoma by indirect pathway suppression.

## Graphical abstract

A cascade‐responsive small‐molecule prodrug (DHU‐NO3) sequentially releases methylene blue (triggered by reactive oxygen species) and nitric oxide (controlled by 405 nm light), enabling precise spatiotemporal coordination of photodynamic therapy and NO gas therapy to effectively inhibit MYC‐amplified osteosarcoma growth.

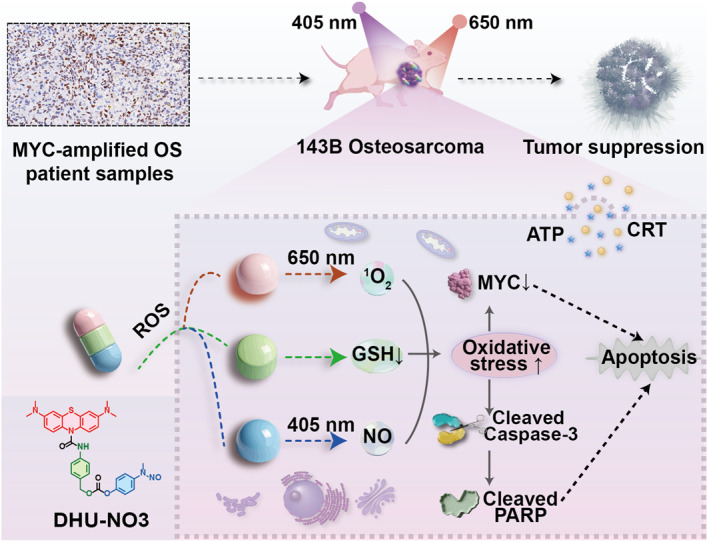

## 1 INTRODUCTION

**FIGURE 1:**
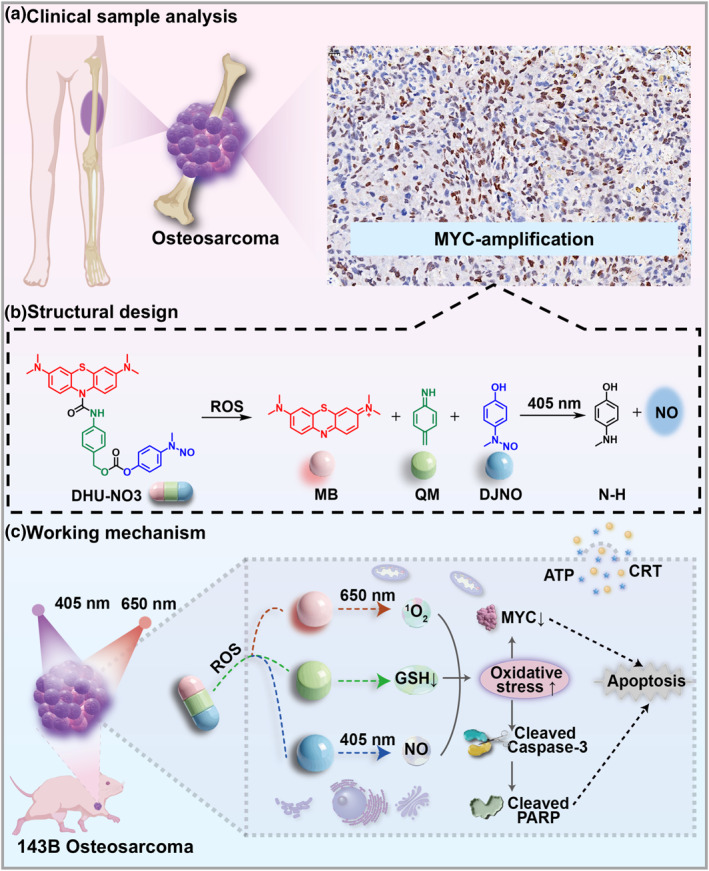
Design and mechanism of a cascade‐responsive theranostic prodrug for MYC‐amplified osteosarcoma. (a) Representative immunohistochemical staining of MYC protein in human osteosarcoma clinical specimens. (b) Rational design and chemical structure of the cascade‐activatable prodrug DHU‐NO3, along with its ROS‐triggered sequential activation pathway. (c) Schematic of the proposed working mechanism.

Osteosarcoma (OS), the most common primary malignant bone tumor in children and adolescents,[^1^, ^2^, ^3^] poses a significant threat due to its highly aggressive and metastatic behavior.[^4^, ^5^] Although surgery combined with neoadjuvant chemotherapy has become the standard of care, long‐term survival rates have improved only marginally over the past 4 decades.[^6^, ^7^, ^8^, ^9^] Accumulating evidence indicates that MYC is frequently amplified and aberrantly overexpressed in OS, and that MYC dysregulation is closely associated with tumor progression and poor clinical outcomes.[^10^, ^11^, ^12^, ^13^, ^14^, ^15^] Consistent with these findings, our histological analysis of clinical specimens confirmed pronounced upregulation of MYC protein in OS tissues, further supporting MYC as a clinically relevant oncoprotein for therapeutic intervention (Figure 1a and Supporting Information S1; Figure S1). However, the MYC protein lacks a well‐defined druggable binding pocket and has therefore long been considered “undruggable” by conventional small‐molecule approaches.[^16^, ^17^] Consequently, most current strategies aim to indirectly modulate MYC activity—by acting on its upstream regulators or downstream effectors.[^18^, ^19^, ^20^, ^21^] This highlights the urgent need for novel therapeutic modalities capable of effectively suppressing MYC‐amplified malignancy.

In this context, there is growing interest in locally targeted therapies that can perturb the tumor microenvironment (TME) and intracellular stress signaling, aiming to overcome the limitations of conventional systemic treatments for MYC‐amplified OS.[^22^, ^23^, ^24^] Photodynamic therapy (PDT) offers a spatially controllable approach in which photosensitizers (PSs) generate reactive oxygen species (ROS) upon light irradiation, leading to oxidative damage.[^25^, ^26^, ^27^, ^28^, ^29^, ^30^] Notably, beyond direct cytotoxicity, PDT has been shown to modulate oncogenic transcription factors,[^31^, ^32^, ^33^, ^34^] including downregulation of c‐MYC expression in specific cancer models, suggesting its potential to disrupt MYC‐driven transcriptional programs.[^35^, ^36^] Concurrently, nitric oxide (NO) is a pleiotropic gaseous signaling molecule whose biological effects are highly dependent on concentration and kinetics. Low, sustained NO levels may promote tumor growth and angiogenesis, whereas high‐dose burst‐like NO delivery can induce DNA damage, mitochondrial dysfunction, and apoptosis.[^37^, ^38^, ^39^, ^40^] Importantly, NO has also been shown to potently modulate MYC transcription and impair its protein stability.[^41^, ^42^, ^43^, ^44^] These complementary features—PDT's capacity to induce oxidative stress and influence signaling pathways, and NO's ability to deliver acute cytotoxic stress and directly inhibit MYC—present a promising therapeutic opportunity. For MYC‐amplified OS, strategic synchronization of on‐demand high‐flux NO release with PDT could generate a synergistic oxidative/nitrosative stress environment. This approach may provide a non‐canonical mechanism to suppress MYC‐associated oncogenic programs without directly targeting the “undruggable” MYC protein.

Notably, OS lesions often exhibit elevated intrinsic oxidative stress, characterized by increased endogenous ROS, which offers a path for tumor‐selective activation of ROS‐responsive prodrugs.[^45^, ^46^] Herein, we tackle a central challenge in combination therapy against MYC‐amplified OS: achieving controllable burst‐like NO release with precise spatiotemporal coupling to PDT in a well‐defined, straightforward system. A temporally activatable, single‐molecule cascade prodrug, DHU‐NO3, was designed and synthesized, based on our prior works utilizing the clinically approved, FDA‐cleared PS methylene blue (MB) as an ROS‐activatable theranostic scaffold.[^47^, ^48^, ^49^, ^50^, ^51^, ^52^, ^53^, ^54^, ^55^] In DHU‐NO3, MB serves as the core theranostic module and is covalently linked—via a quinone methide (QM) self‐immolative spacer—to an *N*‐(4‐hydroxyphenyl)‐N‐methylnitrous amide (DJNO)‐based NO donor (Figure 1b).^[56]^ In the TME, this prodrug follows a well‐orchestrated activation sequence: endogenous ROS first induces linker cleavage and MB release, 405 nm irradiation then elicits on‐demand NO production, and final 650 nm excitation activates MB‐mediated PDT. All this enables precise temporal control over the combined therapeutic regimen. In MYC‐amplified OS cells (143B cell line), efficient internalization and the intended sequential activation of DHU‐NO3—ROS‐triggered MB release, followed by 405 nm‐induced NO production and 650 nm‐activated PDT were observed. Mechanistically, this spatiotemporally coordinated treatment amplified intracellular stress and apoptotic signaling, as evidenced by glutathione (GSH) depletion and adenosine triphosphate (ATP) reduction, upregulation of endoplasmic reticulum (ER) stress and calreticulin (CRT), activation of cleaved caspase‐3 and cleaved poly (ADP‐ribose) polymerase (PARP), and concurrent suppression of MYC‐associated oncogenic programs. Finally, in a 143B OS subcutaneous xenograft model, intratumoral administration of DHU‐NO3 followed by sequential 405 and 650 nm irradiation resulted in robust tumor growth inhibition and a favorable in vivo safety profile (Figure 1c). Collectively, this work establishes a temporally programmable molecular strategy for combining NO and PDT, presenting a promising therapeutic avenue against MYC‐amplified OS.

## 2 RESULTS AND DISCUSSION

### 2.1 Design, synthesis, and photophysical properties

**FIGURE 2:**
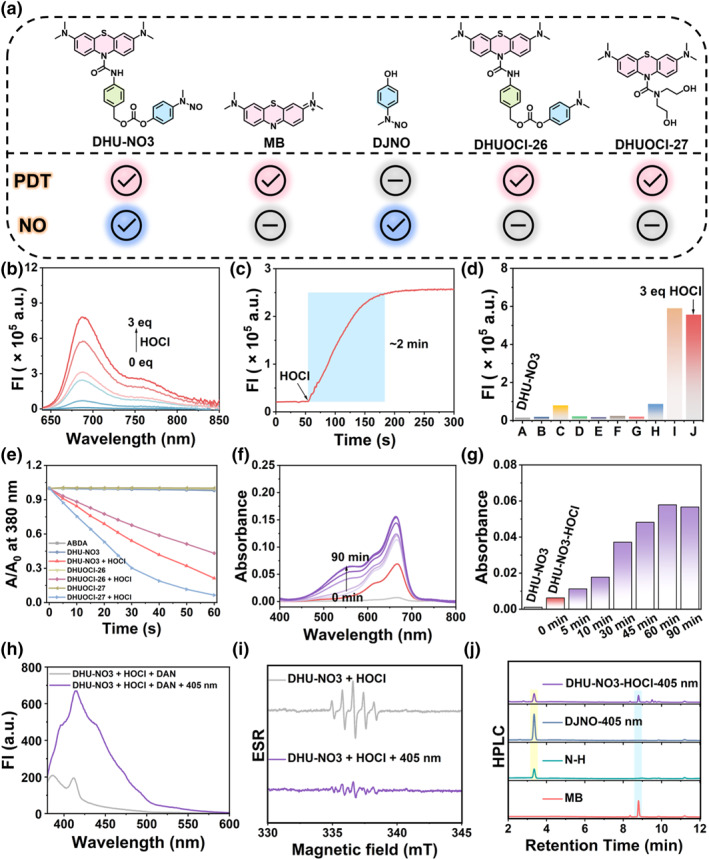
Characterization of reactive oxygen species response activation and controlled nitric oxide (NO) release behavior of DHU‐NO3. (a) Chemical structures of the target prodrug DHU‐NO3 and control compounds (methylene blue, DJNO, DHUOCl‐26, DHUOCl‐27), along with their respective therapeutic effects (photodynamic therapy or NO release). (b) Fluorescence emission spectra (*λ*_ex_ = 620 nm) of DHU‐NO3 (5 μM) in the presence of increasing concentrations of HOCl (0–3 eq). (c) Time‐dependent fluorescence intensity change of DHU‐NO3 (5 μM) at 686 nm after HOCl addition (3 eq, indicated by black arrow). (d) Fluorescence response of DHU‐NO3 (5 μM) to various ROS species (A): DHU‐NO3, (B–I): DHU‐NO3 + H_2_O_2_, ^•^OH, TBHP, ROO^•^, NO, O_2_^−^, *t*‐BuOO^•^, ONOO^−^ (4 eq), (J) DHU‐NO3 + HOCl (3 eq). (e) Degradation of ABDA (100 μM) monitored at 380 nm under 658 nm laser irradiation (20 mW/cm^2^) in the presence of ABDA only, DHU‐NO3 (5 μM) + ABDA, DHU‐NO3 (5 μM) + HOCl (15 μM) + ABDA, DHUOCl‐26 (5 μM) + ABDA, DHUOCl‐26 (5 μM) + HOCl (15 μM) + ABDA, DHUOCl‐27 (5 μM) + ABDA, and DHUOCl‐27 (5 μM) + HOCl (15 μM) + ABDA. The degradation of ABDA followed pseudo‐first‐order kinetics, and the rate constants (k) were obtained by linear fitting of $\ln\left(A_{0}/A_{t}\right)$ versus irradiation time. (f) Absorption spectral changes of the Griess reagent assay monitoring NO release from DHU‐NO3 (pre‐treated with HOCl) under 405 nm LED irradiation (34 mW cm^−2^). (g) Quantitative analysis of NO release kinetics measured at 550 nm from the Griess assay shown in (f). (h) Fluorescence spectral changes of the DAN probe (*λ*_ex_ = 360 nm), confirming light‐triggered NO release from DHU‐NO3 (pre‐treated with HOCl) under 405 nm irradiation. (i) Electron spin resonance spectra of an aqueous solution containing PTIO (10 μM) and HOCl‐pre‐treated DHU‐NO3 (30 μM) before and after 405 nm irradiation. (j) High‐performance liquid chromatography analysis of reaction products.

To achieve on‐demand burst‐like NO release spatiotemporally synchronized with PDT within a single, well‐defined molecular entity—thereby minimizing formulation complexity and ensuring coordinated activation, the prodrug DHU‐NO3 was engineered to satisfy the following three critical requirements. (i) An NO‐releasing module that remains essentially inert under physiological conditions but can be rapidly activated by an external stimulus, thereby preventing prolonged low‐level NO exposure; (ii) a disease‐specific trigger (ROS) to make sure its activation in the TME; and (iii) an integrated redox‐modulating component capable of depleting intracellular GSH, weakening the cellular antioxidant defense, and amplifying PDT‐induced oxidative stress. As illustrated in Figure 1b, DHU‐NO3 adopts a modular architecture: a DJNO moiety serves as the photo‐responsive NO‐donor, enabling controlled burst release of NO upon 405 nm irradiation; a QM‐based self‐immolative linker acts as both a ROS‐responsive activation switch and a GSH‐depleting amplifier; and MB functions as the central theranostic module, providing PDT activity under 650 nm light irradiation along with intrinsic fluorescence for real‐time tracking. To delineate the contribution of each therapeutic component, control compounds DHUOCl‐26 and DHUOCl‐27, which retain ROS‐triggered MB release and PDT capability but lack NO‐release functionality, were synthesized alongside DJNO as a free NO‐donor control (Figure 2a and Supporting Information S1; Scheme S1). The structures of all target molecules and key intermediates were unambiguously confirmed by ^1^H NMR, ^13^C NMR, and high‐resolution mass spectrometry. Complete synthetic routes and characterization data are provided in the SI (Supporting Information S1; Figures S31–S48).

The ROS‐activation of DHU‐NO3 was evaluated under simulated physiological conditions using hypochlorous acid (HOCl) as a representative ROS. The addition of HOCl to a solution of DHU‐NO3 induced concentration‐dependent increases in absorbance at 664 nm (Supporting Information S1; Figure S2) and fluorescence intensity at 686 nm (Figure 2b), unequivocally confirming the successful release of MB. This fluorescence turn‐on response was rapid, reaching completion within 2 min (Figure 2c), demonstrating the high sensitivity and fast activation kinetics. Furthermore, DHU‐NO3 exhibited robust responsiveness to HOCl across the physiologically relevant pH range (Supporting Information S1; Figure S3), ensuring reliable performance under diverse biological conditions. Given that a single ROS may not fully represent the complex TME, broad reactivity toward multiple ROS is likely advantageous for efficient prodrug activation. Indeed, in addition to HOCl, DHU‐NO3 also responded effectively to other biologically relevant ROS, including *t*‐BuOO•, •OH and ONOO^−^, demonstrating broad‐spectrum ROS reactivity while maintaining stability against various biological interferents such as amino acids and ions (Figure 2d and Supporting Information S1; Figure S4). In the selectivity assay, a fluorescence response toward Cu^2+^ was observed only at a supraphysiological concentration of 250 μM (Supporting Information S1; Figure S4E). To further evaluate this potential interference, we performed additional dose‐dependent experiments using lower Cu^2+^ concentrations ranging from 0 to 20 μM. As shown in Supporting Information S1; Figure S5, no obvious fluorescence enhancement was detected under these conditions, whereas HOCl induced a pronounced fluorescence response. Considering that physiological levels of free Cu^2+^ are generally within the low nM to μM range, the interference from Cu^2+^ is expected to be negligible under biologically relevant conditions. We also examined the potential influence of intracellular thiols, including GSH and cysteine (Cys). DHU‐NO3 alone showed no detectable fluorescence response toward either GSH or Cys. Importantly, upon addition of HOCl, a clear fluorescence enhancement was still observed even in the presence of GSH or Cys (Supporting Information S1; Figure S6). These results suggest that DHU‐NO3 retains a reliable ROS‐responsive fluorescence behavior in complex biological redox environments, supporting its suitability for further biological applications. The control compounds DHUOCl‐26 and DHUOCl‐27 exhibited similar ROS‐responsive fluorescence turn‐on behavior (Supporting Information S1; Figures S7–S10), validating their suitability as appropriate controls for independent evaluation of PDT effects.

In addition, the chemical stability of DHU‐NO3 and control compounds was evaluated under physiological conditions. As shown in Supporting Information S1; Figure S11, no significant increase in absorbance at 664 nm was observed for any compound after 24 h incubation in PBS or DMEM containing 10% FBS, indicating the absence of non‐specific hydrolysis or premature MB release. Together with the ROS‐responsive behavior described above, these stability data support the feasibility of DHU‐NO3 activation in the complex tumor environment. The PDT efficacy was assessed using 9, 10‐anthracenediyl‐bis(methylene) dimalonic acid (ABDA) as a singlet oxygen (^1^O_2_) indicator. Control experiments showed negligible ABDA degradation when either ABDA or DHU‐NO3 was irradiated at 658 nm (20 mW/cm^2^). After HOCl pretreatment, DHU‐NO3 efficiently catalyzed ABDA degradation (Figure 2e and Supporting Information S1; Figure S12), confirming its potent ^1^O_2_‐generating capacity. Quantitative comparison of ^1^O_2_ generation efficiency gave ABDA decomposition rate constants of 0.0186 s^−1^ for DHU‐NO3, 0.0125 s^−1^ for DHUOCl‐26, and 0.0315 s^−1^ for DHUOCl‐27 (Supporting Information S1; Figures S13 and S14). These data confirm that all three compounds generate ^1^O_2_ upon activation.

To enable precise spatiotemporal control, the photo‐regulated NO release behavior of DHU‐NO3 was systematically investigated. A solution of DHU‐NO3, pre‐treated with HOCl for 5 min, was exposed to 405 nm LED irradiation (34 mW cm^−2^) for varying durations (Figure 2f,g). The released NO was quantified using the Griess reagent assay by monitoring absorbance at 550 nm, with concentrations determined from a sodium nitrite standard curve. This analysis revealed a cumulative NO release efficiency of approximately 42% after 1 h of irradiation (Supporting Information S1; Figure S15). Notably, 405 nm irradiation further promoted MB release, compensating for any incomplete activation during the initial HOCl‐triggered phase. The NO release was additionally validated using the fluorescent probe 2, 3‐diaminonaphthalene (DAN), which exhibited a marked increase in fluorescence intensity upon 405 nm irradiation (Figure 2h). Moreover, electron spin resonance spectroscopy provided direct evidence of NO radical generation. The disappearance of the characteristic signal of the NO‐trapping agent 2‐phenyl‐4, 4, 5, 5‐tetramethylimidazolineoxyl‐1‐oxyl‐3‐oxide (PTIO) after 405 nm irradiation confirmed NO production (Figure 2i). Crucially, the NO release kinetics remained largely unaffected by environmental pH (Supporting Information S1; Figure S16), underscoring consistent and reliable performance across diverse physiological conditions.

Furthermore, effective NO release was also achieved at lower irradiation power densities (Supporting Information S1; Figure S17), which may enhance biosafety in potential applications. Parallel control experiments with the free NO donor DJNO confirmed its expected photo‐responsive release behavior under identical conditions (Supporting Information S1; Figure S18). High‐performance liquid chromatography analysis verified the formation of the corresponding product N‐H post‐irradiation (Figure 2j). To further confirm the generation of the reactive QM intermediate, which is responsible for GSH depletion, thiol‐trapping experiments were performed using GSH and cysteine (Cys) followed by LC‐MS analysis. As shown in Supporting Information S1 and Figure S19, the QM‐GSH adduct was detected with a measured m/z of 413.75 ([M + H]^+^ calcd for C_17_H_25_N_4_O_6_S^+^: 413.15). Similarly, the QM‐Cys adduct was observed at m/z 227.10 ([M + H]^+^ calcd for C_10_H_15_N_2_O_2_S^+^: 227.08) (Supporting Information S1; Figure S20). These data provide direct chemical evidence for the formation of the QM intermediate upon ROS‐triggered activation of DHU‐NO3. Collectively, these results demonstrate that DHU‐NO3 operates through a sequential activation mechanism involving ROS‐triggered MB release, light‐controlled NO generation, and irradiation‐induced PDT, establishing a robust platform for precise and synergistic anticancer therapy.

### 2.2 Cellular uptake and activation

**FIGURE 3:**
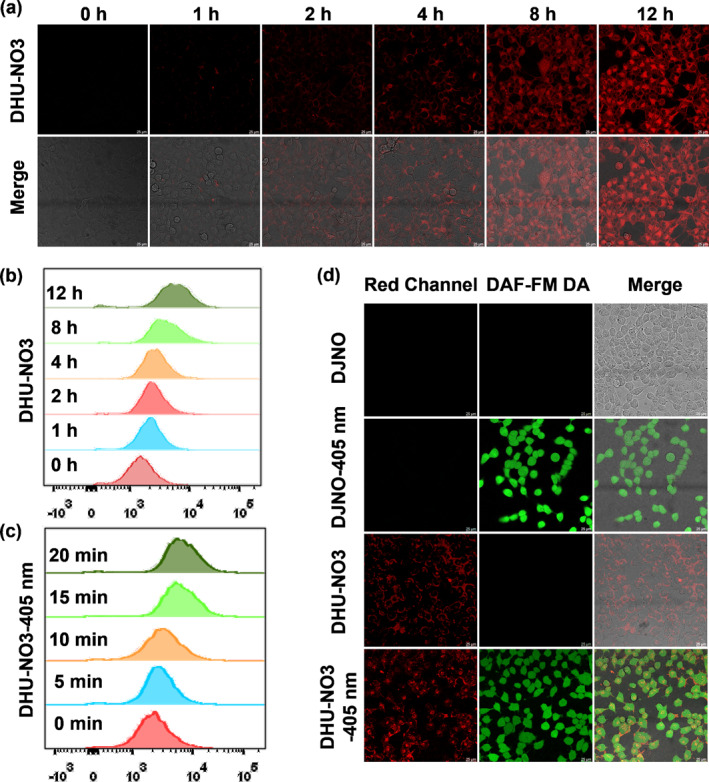
Cellular uptake, activation, and spatiotemporally controlled nitric oxide (NO) release of DHU‐NO3 in 143B OS cells. (a) Confocal laser scanning microscopy (CLSM) images of cells incubated with DHU‐NO3 (10 μM) for different time periods (scale bar: 25 μm). (b) Flow cytometric analysis of fluorescence intensity in cells incubated with DHU‐NO3 (10 μM) for varying durations. (c) Flow cytometric detection of NO release after 405 nm (22 mW cm^−2^) LED irradiation for different time periods. (d) Detection of intracellular NO generation from DJNO (10 μM) and DHU‐NO3 (10 μM) using DAF‐FM DA probe with or without 405 nm irradiation (22 mW cm^−2^, 20 min, scale bar: 25 μm).

Building upon the promising in vitro results, the cascade‐responsive behavior of DHU‐NO3 in 143B OS cells was investigated, beginning with its cellular uptake and endogenous activation. Confocal laser scanning microscopy images revealed a time‐dependent increase in red channel fluorescence intensity for DHU‐NO3 (Figure 3a), as well as for the reference compounds DHUOCl‐26 (Supporting Information S1; Figure S21) and DHUOCl‐27 (Supporting Information S1; Figure S22), over a 12 h incubation period without exogenous stimulation. These observations indicate effective cellular internalization and subsequent intracellular ROS‐triggered activation of the prodrug. Flow cytometry analysis further supported these findings, showing significantly higher fluorescence intensity in DHU‐NO3‐treated cells compared to control groups (Figure 3b).

The photoinduced NO generation was assessed using the specific fluorescent probe 4‐amino‐5‐methylamino‐2′, 7′‐difluorofluorescein diacetate (DAF‐FM DA). Cells pretreated with DHU‐NO3 were irradiated with a 405 nm LED (22 mW cm^−2^) for varying durations. Flow cytometry revealed a time‐dependent increase in fluorescence intensity, peaking at approximately 20 min (Figure 3c). This trend was corroborated by Confocal laser scanning microscopy imaging (Figure 3d), in which non‐irradiated cells exhibited minimal background fluorescence, whereas strong green fluorescence was observed after 20 min of irradiation. Collectively, these results demonstrate that DHU‐NO3 sequentially responds to endogenous ROS and exogenous light within cellular environments, enabling spatiotemporally controlled release of MB followed by precise NO generation, thereby elucidating the mechanistic basis for its synergistic therapeutic action.

### 2.3 Synergistic cytotoxicity and mechanism investigation

**FIGURE 4:**
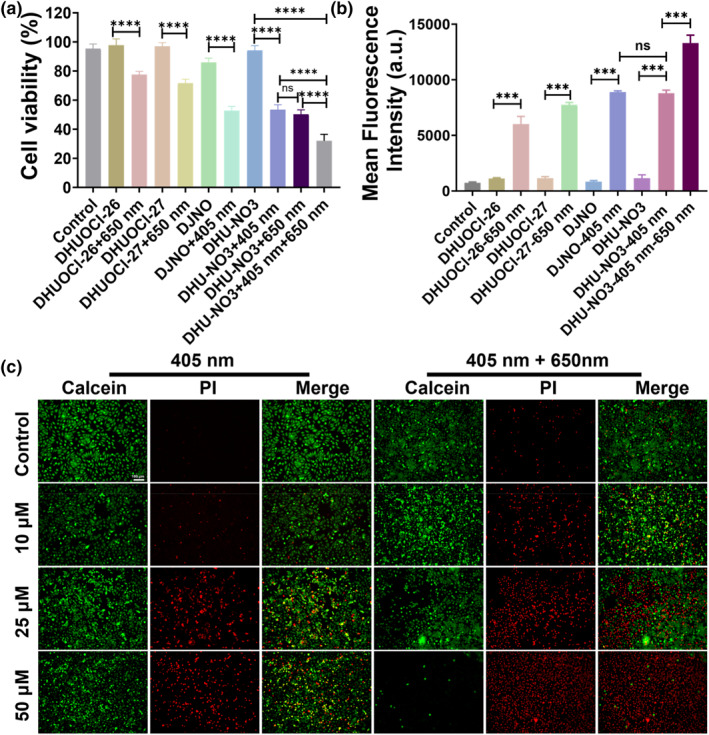
In vitro synergistic photocytotoxicity of DHU‐NO3 in 143B OS cells. (a) Cell viability of 143B OS cells determined by Cell Counting Kit‐8 (CCK‐8) assay after treatment with DHU‐NO3 or control compounds (10 μM) and either kept in the dark or exposed to 405 nm (22 mW cm^−2^, 20 min) or 650 nm (14 mW cm^−2^, 5 min) irradiation. (b) Quantitative analysis of DCF fluorescence intensity. (c) Live/Dead staining of 143B OS cells using Calcein‐AM/PI after incubation with DHU‐NO3 at different concentrations (0 μM, 10 μM, 25 μM, 50 μM), followed by 405 nm irradiation alone or combined 405/650 nm irradiation (scale bar: 100 μm). Error bars denote SD, **p < 0.01, ***p < 0.001, ****p < 0.0001. All quantitative data are presented as mean ± SD. Statistical comparisons were performed using unpaired Student's *t*‐test for two groups or one‐way ANOVA with Tukey's post hoc test for three or more groups (detailed in Supporting Information S1; Section 1.20).

Motivated by these results, the in vitro biocompatibility of DHU‐NO3 was evaluated. The Cell Counting Kit‐8 assay indicated that all compounds (including DHU‐NO3 and control agents) maintained high cell viability after dark incubation, demonstrating low dark toxicity. Figure 4a shows the cytotoxicity of each treatment group at 10 μM (data for other concentrations are presented in Supporting Information S1; Figure S23). Photocytotoxicity was then assessed under light irradiation. Compared to the NO‐only (DJNO + 405 nm) or PDT‐only (DHUOCl‐26/27 + 650 nm) treatment groups, the DHU‐NO3‐treated group exhibited a marked decrease in cell viability under dual‐wavelength (405 nm + 650 nm) irradiation, indicating a synergistic effect combining PDT and NO gas therapy. To determine whether this synergistic treatment is accompanied by ROS generation, intracellular ROS levels were measured using the 2′, 7′‐dichlorodihydrofluorescein diacetate (DCFH‐DA) probe. The results showed negligible fluorescence in non‐irradiated cells, whereas irradiated cells exhibited intense green fluorescence, confirming robust light‐induced ROS production (Figure 4b and Supporting Information S1; Figure S24). Live/Dead staining (Calcein‐AM (calcein acetoxymethyl ester)/PI (propidium iodide)) further visually confirmed the synergistic killing effect: the DHU‐NO3 group under dual‐light irradiation exhibited a substantially higher rate of cell death (Figure 4c and Supporting Information S1; Figure S25).

**FIGURE 5:**
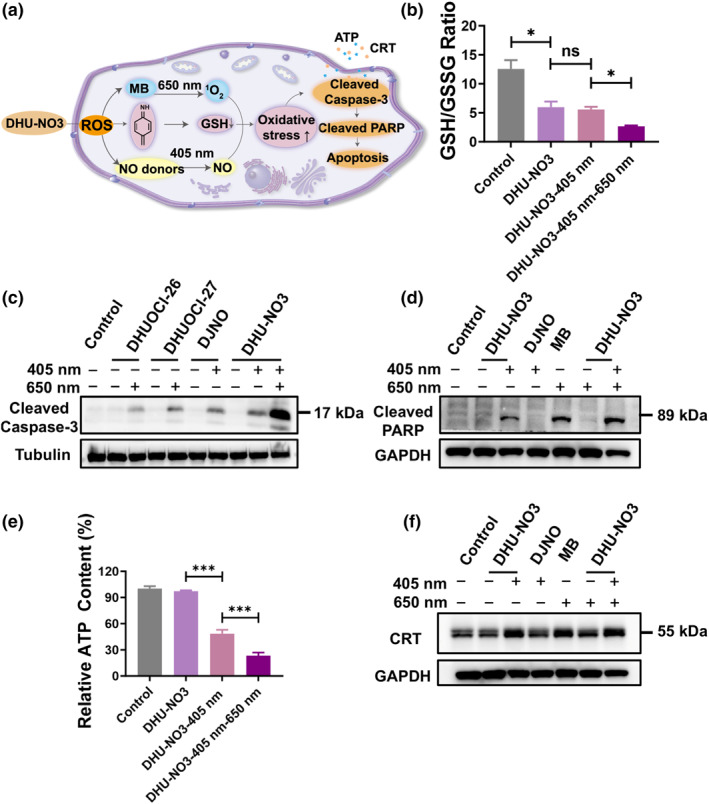
Molecular mechanism of DHU‐NO3‐induced apoptosis in 143B OS cells. (a) Schematic diagram illustrating the proposed mechanism of DHU‐NO3‐induced tumor cell death via combination therapy. (b) Intracellular GSH/GSSG ratio in 143B cells following various treatments: Control, DHU‐NO3, DHU‐NO3 with 405 nm light (22 mW cm^−2^, 20 min), and DHU‐NO3 with sequential 405 nm (22 mW cm^−2^, 20 min) and 650 nm (14 mW cm^−2^, 5 min) irradiation. (c) Western blot (WB) analysis of cleaved caspase‐3 protein expression in 143B cells after different treatments. (d) WB analysis of cleaved PARP protein expression in 143B cells after different treatments. (e) Intracellular adenosine triphosphate (ATP) levels in 143B cells following various treatments. (f) WB analysis of CRT protein expression in 143B cells after different treatments. Error bars represent SD; *p < 0.05, **p < 0.01, ***p < 0.001.

To elucidate the mechanism underlying the observed synergistic cytotoxicity, the multifaceted actions of DHU‐NO3 were systematically investigated at the molecular level (Figure 5a). In addition to the ROS‐responsive release of MB and photo‐controlled generation of NO, the activation process releases a QM derivative—a potent electrophile that depletes intracellular GSH. Quantitative analysis of the GSH/GSSG (glutathione disulfide) ratio confirmed that combination therapy induced the most significant decrease in this ratio, reflecting a marked reduction in intracellular GSH levels (Figure 5b). This QM‐mediated GSH depletion effectively compromises the cellular antioxidant defense system, thereby amplifying the oxidative stress generated by both PDT and high‐dose NO. Whether this enhanced oxidative stress triggers apoptotic cell death was then examined. Western blot (WB) analysis showed that combined treatment with DHU‐NO3 under dual‐wavelength irradiation significantly upregulated key apoptotic regulators, including cleaved caspase‐3 and cleaved poly (ADP‐ribose) polymerase (PARP, Figure 5c,d). The pronounced increase in these proteolytic fragments confirms the superior apoptosis‐inducing capability of this synergistic approach. Concurrently, monitoring of changes in cellular energy metabolism and stress responses revealed a significant decrease in intracellular ATP levels, indicating mitochondrial dysfunction and disruption of energy homeostasis (Figure 5e). Furthermore, WB analysis demonstrated upregulation of CRT, suggesting induction of ER stress (Figure 5f). Full, uncropped WB membranes for all key proteins are provided in Supporting Information S1; Figure S28.

Collectively, these results demonstrate that DHU‐NO3 potently enhances oxidative stress via combined ROS and NO generation, coupled with GSH depletion, disrupts cellular energy metabolism, and induces ER stress. The convergence of these stress pathways effectively activates the apoptotic cascade, ultimately resulting in robust tumor cell death.

### 2.4 Transcriptomics analysis

**FIGURE 6:**
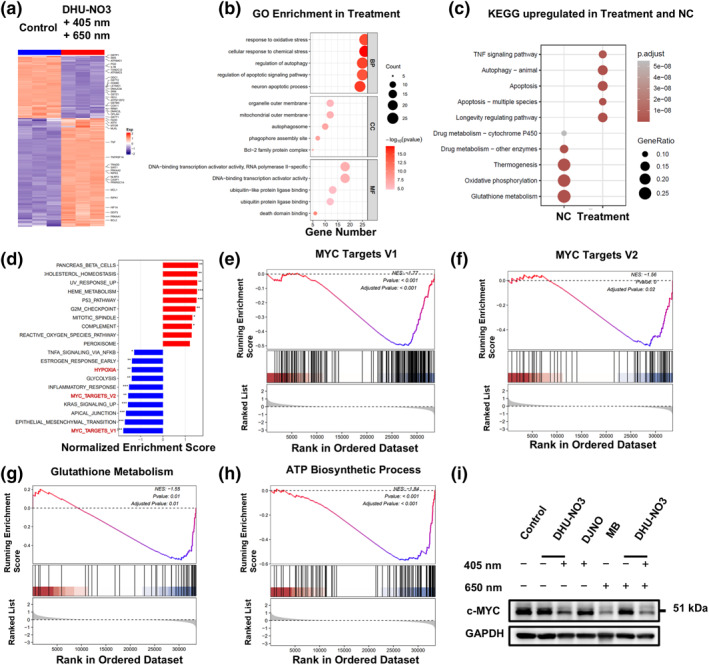
Transcriptomic profiling and pathway analysis of DHU‐NO3 therapy for 143B OS cells. (a) Heat map showing differentially expressed genes (DEGs) between control and DHU‐NO3 therapy groups in 143B cells (*n* = 3). (b) Kyoto encyclopedia of genes and genomes (KEGG) pathway enrichment analysis of DEGs. (c) Gene ontology (GO) enrichment analysis of significantly altered genes in biological process (BP), cellular component (CC), and molecular function (MF) categories. (d) Hallmark gene set enrichment analysis (GSEA) following DHU‐NO3 treatment. (e–h) GSEA of significantly enriched pathways. (i) Western blot (WB) analysis of c‐MYC protein expression under different treatment conditions.

To gain deeper insight into the molecular mechanisms underlying DHU‐NO3 combination therapy, comprehensive transcriptomic profiling was performed using RNA sequencing. Comparative analysis between control and treated groups revealed significant transcriptomic alterations, with 179 upregulated and 103 downregulated genes (Figure 6a and Supporting Information S1; Figure S26). Gene ontology enrichment analysis indicated a fundamental shift in cellular states following treatment. Specifically, cellular activities in the control group were predominantly associated with energy metabolism, including aerobic respiration and oxidative phosphorylation (Supporting Information S1; Figure S27). In contrast, the DHU‐NO3‐treated group showed significant enrichment for biological processes related to the oxidative stress response and regulation of the apoptotic signaling pathway (Figure 6b). Kyoto encyclopedia of genes and genomes pathway analysis further corroborated these findings, demonstrating marked activation of the tumor necrosis factor signaling pathway and apoptotic pathway (Figure 6c). Notably, both Hallmark gene set analysis and gene set enrichment analysis (GSEA) revealed significant suppression of MYC Targets V1 and V2 gene sets, indicating substantial inhibition of the MYC signaling network (Figure 6d–f). This transcriptomic finding was validated at the protein level by WB analysis, which confirmed significant downregulation of c‐MYC expression in treated cells (Figure 6i). Furthermore, GSEA indicated downregulation of additional pathways, including glutathione metabolism and ATP biosynthetic process, consistent with our experimental observations of GSH depletion and ATP reduction (Figure 6g,h). Collectively, these transcriptomic data demonstrate that DHU‐NO3 combination therapy disrupts cellular energy metabolism, amplifies oxidative stress, and suppresses the MYC signaling network, thereby providing a comprehensive molecular explanation for its potent antitumor efficacy.

### 2.5 In vivo antitumor efficacy and biosafety evaluation

**FIGURE 7:**
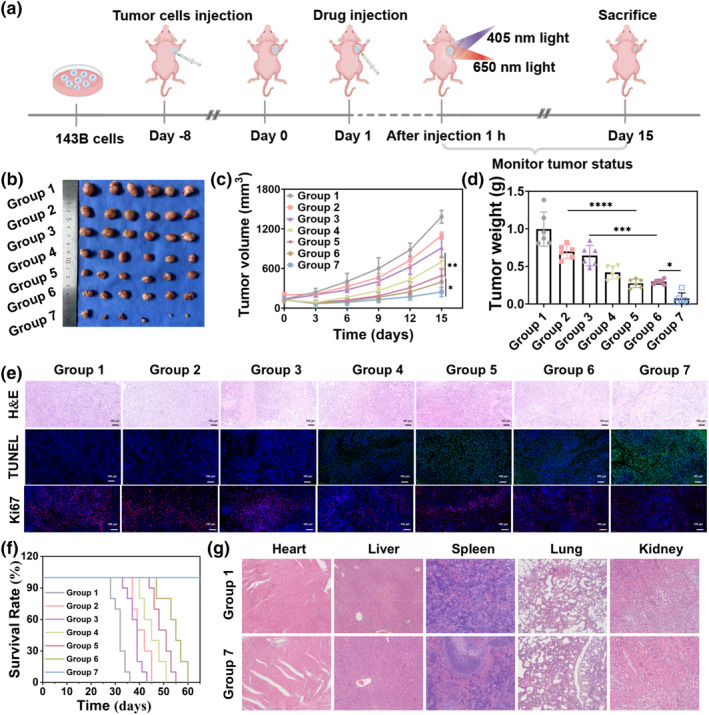
In vivo antitumor efficacy and biosafety evaluation of DHU‐NO3 in a 143B OS model. (a) Schematic illustration of the therapeutic regimen in tumor‐bearing mice. (b) Representative photographs of excised tumors from each treatment group (Group 1: Control; Group 2: methylene blue (MB)) (1 mg/mL, 50 μL); Group 3: DHU‐NO3 (1 mg/mL, 50 μL); Group 4: DJNO (1 mg/mL, 50 μL) + 405 nm; Group 5: MB (1 mg/mL, 50 μL) + 650 nm; Group 6: DHU‐NO3 (1 mg/mL, 50 μL) + 405 nm; and Group 7: DHU‐NO3 (1 mg/mL, 50 μL) + 405 nm + 650 nm (405 nm: 180 mW cm^−2^, 10 min, 650 nm: 300 mW cm^−2^, 5 min) (*n* = 6). (c) Tumor volume growth curves of mice during the treatment period (*n* = 6). (d) Average tumor weights measured at the endpoint of the study (*n* = 6). (e) H&E staining and immunofluorescence staining (transferase dUTP Nick end labeling (TUNEL) and Ki67) of tumor tissue sections from different treatment groups (scale bar: 100 μm). (f) Survival curves of mice in different treatment groups during the study period. (g) H&E staining of major organs (heart, liver, spleen, lung, kidney) from the control group (Group 1) and the DHU‐NO3 + 405 nm + 650 nm (Group 7) combination therapy group (scale bar: 70 μm). Error bars denote SD, *p < 0.05, **p < 0.01, ***p < 0.001, ****p < 0.0001.

Building on the promising in vitro synergistic antitumor effects, the therapeutic efficacy of DHU‐NO3 was evaluated in a subcutaneous xenograft mouse model of 143B OS. The experimental regimen was schematically illustrated in Figure 7a. In vivo fluorescence imaging demonstrated that following intratumoral injection, DHU‐NO3 was specifically activated and effectively accumulated at the tumor site (Supporting Information S1; Figure S29). To comprehensively assess therapeutic outcomes, tumor‐bearing mice were randomly assigned to seven groups (*n* = 6): (1) Control (no treatment); (2) MB; (3) DHU‐NO3; (4) DJNO + 405 nm; (5) MB + 650 nm; (6) DHU‐NO3 + 405 nm; and (7) DHU‐NO3 + 405 nm + 650 nm (405 nm: 180 mW cm^−2^, 10 min; 650 nm: 300 mW cm^−2^, 5 min). Tumor volume and weight were monitored throughout the treatment period (Figure 7c). After 14 days, the control group exhibited rapid tumor progression. In contrast, tumor growth in the combination therapy group (DHU‐NO3 + 405 nm + 650 nm) was most effectively suppressed, demonstrating statistically significant efficacy compared to either PDT or NO monotherapy. Subsequent examination of excised tumors by weight measurement and photography confirmed that tumors in the DHU‐NO3 combination therapy group were substantially smaller and lighter than those in all other groups. Histological analyses further validated the therapeutic effects (Figure 7b,d). Hematoxylin and eosin (H&E) staining of tumor sections revealed extensive regions of apoptosis and necrosis in the combination therapy group (Figure 7e). Additionally, immunohistochemical staining for Ki67 showed a marked reduction in proliferating cells, and the terminal deoxynucleotidyl transferase dUTP Nick end labeling assay confirmed widespread cellular apoptosis (Figure 7e).

The biosafety of the treatments was systematically evaluated. Throughout the study, the body weights of mice in all groups remained stable, with no significant weight loss observed (Supporting Information S1; Figure S30). Notably, survival analysis revealed a 100% survival rate in the DHU‐NO3 combination therapy group, with no mortality recorded over the entire 60‐day monitoring period (Figure 7f), representing the most favorable outcome among all experimental groups. Furthermore, histological examination of major organs (heart, liver, spleen, lung, and kidney) via H&E staining showed no apparent pathological abnormalities in either the control or combination therapy group (Figure 7g). Collectively, these findings confirm the favorable biocompatibility of DHU‐NO3 in vivo and support its potent antitumor efficacy against 143B OS.

## 3 CONCLUSION

In summary, we report the successful development of DHU‐NO3, a novel small‐molecule cascade‐activatable prodrug that integrates sequential ROS‐responsive and photo‐controllable functionalities within a single chemical framework. The activation proceeds through a three‐step cascade: ROS‐triggered release of the PS MB, 405 nm light‐induced NO generation, and 650 nm laser‐initiated PDT, enabling spatiotemporally precise synergistic treatment. Mechanistically, this multimodal approach potently induces apoptosis, as demonstrated by upregulation of cleaved caspase‐3 and cleaved PARP, and concurrently triggers ER stress. Transcriptomic analysis and WB validation further revealed that the treatment significantly suppressed the MYC oncogenic signaling network, underscoring a key molecular mechanism underlying its therapeutic efficacy. In vivo, DHU‐NO3 mediates remarkable tumor growth inhibition in a 143B OS xenograft model, with no observable systemic toxicity. Although the present study provides promising proof‐of‐concept evidence, further optimization may help to broaden the potential applications of this platform. For example, the development of systemic delivery strategies and validation in orthotopic bone tumor models would enable a more clinically relevant evaluation of therapeutic performance. In addition, extending the activation wavelength through rational optimization of the NO‐donor structure may further improve its applicability to deep‐seated tumors. Despite these aspects requiring further investigation, this work establishes a new molecular design paradigm for precision combination therapy in osteosarcoma and offers a feasible chemical biology strategy for indirectly downregulating the traditionally “undruggable” oncoprotein MYC.

## CONFLICT OF INTEREST STATEMENT

The authors declare no conflicts of interest.

## ETHICS STATEMENT

Our clinical sample study was approved by the Institutional Research Ethics Committee of Shanghai General Hospital, Shanghai Jiao Tong University School of Medicine (Approval No. 2021KY103). All procedures for consideration of animal welfare were reviewed and approved by the Animal Care and Use Committee of the Shanghai General Hospital (2025AW055).

## Supporting information

Supporting Information S1

## Data Availability

The data that supports the findings of this study are available in the supplementary material of this article.
