# Supplementary material for: A single‐molecule prodrug synergistically suppresses MYC‐amplified osteosarcoma through sequential nitric oxide release and photodynamic therapy
Source: Smart Mol. 2026 Aug 3:e70084. Online ahead of print. doi: 10.1002/smo2.70084 (PMC13430155; doi:10.1002/smo2.70084)
Supplement: Supplementary file 1 — Supporting Information S1 [file SMO2-9999-0-s001.docx]

Supporting Information

A single-molecule prodrug synergistically suppresses MYC-amplified osteosarcoma through sequential nitric oxide release and photodynamic therapy

**Shuxin Peng^1^, Jiangpeng Wu^2^, Shasha Wang^1^, Ying Gao^1^, Xuran Guo^2^, Mengpan Li^2^, Youjin Wu^1^, Yun Wang^2^, Zhengdong Cai^2^, Yingqi Hua^2,3,^*, Peng Wei^1,^*, Yinghua Gao^2,^*, Tao Yi^1,^***

**1 Experimental Section**

**1.1 Materials and Instrumentation**

All solvents and reagents were of analytical grade and used without further purification unless otherwise specified. Methylene Blue (MB), sodium carbonate (Na_2_CO_3_), sodium dithionite (Na_2_S_2_O_4_), hydrochloric acid (HCl), hydrogen peroxide (H_2_O_2_), triphosgene (BTC), 4-(dimethylamino)phenol, 4-aminobenzyl alcohol, 4-nitrophenyl chloroformate, sodium nitrite (NaNO_2_), sodium chloride (NaCl), diethanolamine, 4-methylaminophenol sulfate, 4-dimethylaminopyridine (DMAP), acetic acid, pyridine, triethylamine (Et_3_N) potassium superoxide (KO_2_), ferrous sulfate (FeSO_4_), sodium hydroxide (NaOH), dimethylformamide (DMF), manganese dioxide (MnO_2_), 2-phenyl-4, 4, 5, 5-tetramethylimidazolineoxyl-1-oxyl-3-oxide (PTIO), 2, 2’-azobis(2-amidinopropane) dihydrochloride (AAPH), 2, 3-diaminonaphthalene (DAN) and 9, 10-bis-(methylene) dimalonic acid (ABDA) were obtained from commercial suppliers. Cell culture reagents, including phosphate-buffered saline (PBS), Dulbecco’s modified Eagle’s medium (DMEM), trypsin, penicillin/streptomycin, and fetal bovine serum (FBS), were obtained from Gibco (Shanghai, China). Cell counting Kit-8, calcein-AM/ propidium iodide (PI) double stain kit, GSH and GSSG assay kit, 2’, 7’-dichlorodihydrofluorescein diacetate (DCFH-DA), and enhanced ATP assay kit were purchased from Beyotime Biotechnology (Shanghai, China). CRT Rabbit Monoclonal Antibody CRT (ab92516), Cleaved PARP (ab278604), Cleaved Caspase 3 (ab32042), and c-MYC (ab32072).

^1^H and ^13^C NMR spectra were recorded on a Bruker AV400 MHz NMR spectrometer using CDCl_3_ or DMSO-*d*_6_ as solvents, with chemical shifts reported in parts per million (ppm) relative to tetramethyl silane (TMS) as an internal standard. High-resolution mass spectra (HRMS) were acquired on a Bruker Micro TOF II instrument equipped with an electrospray ionization (ESI) source. UV-vis absorption spectra were measured using a Shimadzu UV-2600 spectrophotometer. Steady-state fluorescence spectra were obtained at room temperature on an Edinburgh Instruments FLS1000 spectrometer using a xenon lamp as the excitation source. High-Performance Liquid Chromatography (HPLC) was performed with an Agilent Technologies 1200 series system. Electron spin resonance (ESR) spectra were recorded on a Magnettech ESR5000 spectrometer at room temperature. Confocal laser scanning microscopy (CLSM) images were captured using a Leica SP8 system. In vivo fluorescence imaging was performed using an IVIS Lumina III small-animal imaging system.

**1.2 Synthesis of the Molecules**

**Scheme S1:** Synthesis process of compounds.

**Synthesis of Compound DJNO.** 4-(Methylamino)phenol sulfate (1.8 g, 10.0 mmol, 1 eq) and 50 mL of acetic acid were added to a 250 mL three-necked flask. Subsequently, a solution of NaNO_2_ (3.5 g, 50.0 mmol, 5 eq) in 10 mL of deionized water was added dropwise to the above mixture. The reaction was carried out in the dark for 3 h, then quenched with 50 mL of water. The pH was adjusted to 7.0 using sodium bicarbonate. The mixture was extracted with ethyl acetate, and the combined organic layers were dried. The crude product was purified by column chromatography (petroleum ether/ethyl acetate = 4: 1, v/v) to afford 0.36 g of compound DJNO as a pale-yellow solid in 23.9% yield.

^1^H NMR (400 MHz, DMSO-*d*_6_, δ): 9.77 (s, 1H), 7.44 - 7.36 (m, 2H), 6.92 - 6.85 (m, 2H), 3.37 (t, *J* = 2.0 Hz, 3H). ^13^C NMR (100 MHz, DMSO-*d*_6_, δ): 157.0, 134.0, 121.8, 115.8, 32.5. HRMS (ESI) *m*/*z*: [M + H]^+^ calcd for C_7_H_9_N_2_O_2_^+^, 153.0659; found, 153.0655.

**Synthesis of Compound FDOCl-2.** Compound FDOCl-2 was synthesized according to our reported method.^[1]^

**Synthesis of Compound 1-1.** To a solution of 4-aminobenzyl alcohol (0.3 g, 2.4 mmol, 1 eq) and pyridine (0.2 g, 2.4 mmol, 1 eq) in dichloromethane (DCM, 20 mL), FDOCl-2 (1.0 g, 2.9 mmol, 1.2 eq) was added gradually. The reaction mixture was stirred at 40°C for 12 h and monitored by TLC. After cooling to room temperature, the mixture was diluted with DCM (3 × 100 mL) and washed with brine (100 mL). The organic layer was dried over anhydrous Na_2_SO_4_, concentrated under reduced pressure, and purified by silica gel column chromatography to yield a pale blue solid, compound 1-1 (0.4 g, 41.3% yield).

^1^H NMR (400 MHz, DMSO-*d*_6_, δ): 8.06 (s, 1H), 7.44 - 7.36 (m, 4H), 7.18 (d, *J* = 8.5 Hz, 2H), 6.75 (d, *J* = 2.8 Hz, 2H), 6.69 (dd, *J* = 9.0, 2.8 Hz, 2H), 4.41 (s, 2H), 2.90 (s, 12H). ^13^C NMR (100 MHz, DMSO-*d*_6_, δ): 152.8, 148.5, 138.0, 136.4, 133.1, 128.1, 126.9, 126.5, 119.7, 111.1, 110.3, 62.5, 40.1. HRMS (ESI) *m*/*z*: [M + H]^+^ calcd for C_24_H_27_N_4_O_2_S^+^, 435.1849; found, 435.1847.

**Synthesis of Compound 1-2.** A mixture of compound 1-1 (0.5 g, 1.2 mmol, 1 eq), DMAP (0.2 g, 1.2 mmol, 1 eq), and 4-nitrophenyl chloroformate (0.3 g, 1.4 mmol, 1.2 eq) in DCM (20 mL) was cooled to 0°C. Et_3_N (0.3 g, 3.5 mmol, 3 eq) was added dropwise. The resulting mixture was stirred at 0°C for 30 min, warmed to room temperature, and stirred for an additional 5 h. The reaction was quenched with water (50 mL) and extracted with DCM (3 × 50 mL). The combined organic layers were washed with brine (20 mL), dried over Na_2_SO_4_, and concentrated under reduced pressure. Purification by silica gel column chromatography (petroleum ether/ethyl acetate = 3: 1 v/v) afforded compound 1-2 as a pale green powder (0.4 g, 89.0% yield).

^1^H NMR (400 MHz, DMSO-*d*_6_, δ): 8.36 - 8.15 (m, 3H), 7.59 - 7.54 (m, 1H), 7.53 - 7.46 (m, 2H), 7.41 (dd, *J* = 8.9, 2.3 Hz, 2H), 7.34 (d, *J* = 8.3 Hz, 2H), 7.21 (d, *J* = 9.3 Hz, 1H), 6.75 (t, *J* = 2.4 Hz, 2H), 6.72 - 6.65 (m, 2H), 5.20 (d, *J* = 20.0 Hz, 2H), 2.91 (s, 12H). ^13^C NMR (100 MHz, DMSO-*d*_6_, δ): 163.6, 155.2, 152.7, 151.9, 148.5, 145.1, 140.8, 140.2, 139.7, 133.0, 129.4, 129.1, 128.3, 128.1, 128.0, 127.9, 126.9, 125.8, 125.3, 124.1, 122.5, 119.8, 119.8, 115.3, 111.1, 110.3, 70.0, 40.1. HRMS (ESI) *m*/*z*: [M + H]^+^ calcd for C_31_H_30_N_5_O_6_S^+^, 600.1912; found, 600.1912.

**Synthesis of DHU-NO3.** To a 100 mL single-necked round-bottom flask were added compound 1-2 (0.6 g, 1.0 mmol, 1 eq) and DCM (20 mL), and the mixture was stirred until complete dissolution. Et_3_N (0.3 g, 3.0 mmol, 3 eq), DMAP (0.1 g, 1.0 mmol, 1 eq), and compound DJNO (0.2 g, 1.5 mmol, 1.5 eq) were sequentially introduced to the reaction mixture. The solution was stirred continuously for 4 h. The reaction mixture was extracted with DCM (3 × 100 mL). The organic phase was dried over Na_2_SO_4_, concentrated under reduced pressure, and purified by flash silica gel chromatography (petroleum ether/ethyl acetate = 3: 1, v/v) to obtain DHU-NO3 as a pale blue powder (0.2 g, 26.0% yield).

^1^H NMR (400 MHz, DMSO-*d*_6_, δ): 8.27 (s, 1H), 7.72 - 7.66 (m, 2H), 7.54 - 7.48 (m, 2H), 7.45 - 7.39 (m, 4H), 7.37 - 7.32 (m, 2H), 6.76 (d, *J* = 2.8 Hz, 2H), 6.69 (dd, *J* = 9.0, 2.8 Hz, 2H), 5.21 (s, 2H), 3.43 (s, 3H), 2.91 (s, 12H). ^13^C NMR (100 MHz, CDCl_3_, δ): 153.4, 153.0, 150.0, 149.1, 140.0, 139.5, 134.5, 129.7, 128.9, 127.1, 122.2, 120.1, 119.2, 111.3, 111.1, 70.5, 40.7, 31.5. HRMS (ESI) *m*/*z*: [M + H]^+^ calcd for C_32_H_33_N_6_O_5_S^+^, 613.2228; found, 613.2226.

**Synthesis of FDOCl-26.** To a solution of compound 1-2 (0.6 g, 1.0 mmol, 1 eq) in CH_2_Cl_2_ (20 mL) was added Et_3_N (0.3 g, 3.0 mmol, 3 eq), DMAP (0.1 g, 1.0 mmol, 1 eq), and 4-(dimethylamino) phenol (0.2 g, 1.5 mmol, 1.5 eq). The mixture was stirred for 4 h at room temperature, then diluted with CH_2_Cl_2_ (3 × 50 mL). After washing with saturated brine (20 mL), the organic phase was dried with anhydrous sodium sulfate (Na_2_SO_4_). The product was separated and collected by silica gel column chromatography (petroleum ether/ethyl acetate = 3: 1, v/v), then concentrated by rotary evaporation, yielding the target compound DHUOCl-26 as a pale blue powder (75.0 mg, 12.5% yield).

^1^H NMR (400 MHz, CDCl_3_, δ): 7.47 - 7.39 (m, 4H), 7.32 (d, *J* = 8.2 Hz, 2H), 7.04 - 6.98 (m, 3H), 6.73 (d, *J* = 2.8 Hz, 2H), 6.71 - 6.64 (m, 4H), 5.16 (s, 2H), 2.96 (s, 12H), 2.92 (s, 6H). ^13^C NMR (100 MHz, CDCl_3_, δ): 154.5, 153.1, 149.3, 148.9, 139.3, 134.6, 129.7, 129.4, 127.8, 127.2, 121.5, 119.2, 113.2, 111.4, 111.1, 70.1, 41.1, 40.8. HRMS (ESI) *m*/*z*: [M + H]^+^ calcd for C_33_H_36_N_5_O_4_S^+^, 598.2483; found, 598.2483.

**Synthesis of FDOCl-27.** Compound FDOCl-2(1.0 g, 2.9 mmol, 1 eq) was dissolved in 20 mL of DCM in an ice bath. Diethanolamine (0.4 g, 3.5 mmol, 1.2 eq) dissolved in 10 mL of CH_2_Cl_2_ was slowly added to the reaction mixture, which was stirred for 4 h. Upon completion, the mixture was diluted with DCM (100 mL) and washed with brine. The organic phase was dried over anhydrous Na_2_SO_4_ and concentrated under reduced pressure. The crude product was purified by silica gel column chromatography (eluent: petroleum ether/ethyl acetate = 1: 4, v/v) to afford DHUOCl-27 as a pale blue powder (0.5 g, 44.2% yield).

^1^H NMR (400 MHz, DMSO-*d*_6_, δ): 7.40 (d, *J* = 8.9 Hz, 2H), 6.65 (d, *J* = 2.8 Hz, 2H), 6.59 (dd, *J* = 8.9, 2.9 Hz, 2H), 4.65 (t, *J* = 5.1 Hz, 2H), 3.41 (q, *J* = 5.9 Hz, 4H), 3.23 (t, *J* = 6.1 Hz, 4H), 2.85 (s, 12H). ^13^C NMR (100 MHz, DMSO-*d*_6_, δ): 157.9, 147.7, 131.3, 128.4, 121.8, 111.4, 110.6, 58.9, 50.9, 40.3. HRMS (ESI) *m*/*z*: [M + H]^+^ calcd for C_21_H_29_N_4_O_3_S^+^, 417.1955; found, 417.1957

**1.3 Preparation of probes and different analytes**

The Stock solutions of DHU-NO3, DHUOCl-26, DHUOCl-27, and DJNO (5 mM) were prepared in DMF and diluted in PBS buffer (10 mM, pH 7.4) to the desired test concentrations. The compounds were thoroughly mixed with various analytes for 30 min, followed by measurement of fluorescence or absorption spectra using a fluorescence or UV-vis spectrophotometer. Unless otherwise specified, all fluorescence measurements for MB were conducted with an excitation wavelength of 620 nm, with emission spectra collected from 640 nm to 850 nm, and the maximum emission recorded at 686 nm.

Different analytes were prepared in ddH_2_O according to our reported procedure, and the details are as follows. HOCl was obtained from NaOCl solution (0.1 M). H_2_O_2_ was diluted from a 30 % solution. ^•^OH (Hydroxyl radical) was generated by the Fenton reaction (H_2_O_2_: FeSO_4_ = 1:10), and the concentration of ^•^OH was equal to the concentration of H_2_O_2_. TBHP (tert-butyl hydroperoxide) was obtained from 70% TBHP solution in ddH_2_O. ROO^•^ (Peroxyl radical) was prepared by dissolving 2, 2’-azobis(2-amidinopropane) dihydrochloride in ddH_2_O. NO was prepared by dissolving SNP (sodium nitroferricyanide (III) dihydrate) in ddH_2_O. O_2_⁻ (Superoxide anion) was prepared by dissolving KO_2_ (potassium superoxide) in DMSO. *t*-BuOO^•^ was prepared by adding TBHP in the presence of 10 equiv. of FeSO_4_ and the concentration of *t*-BuOO^•^ was equal to the TBHP concentration.

ONOO^−^ (Peroxynitrite): H_2_O_2_ and HCl solution were added to the reaction vessel and stirred thoroughly. NaNO_2_ was then rapidly introduced, resulting in an immediate yellow solution, followed within 1 s by the addition of NaOH solution to maintain the yellow color. MnO_2_ is subsequently added to remove excess hydrogen peroxide. The concentration is determined via UV-vis spectrophotometric analysis by measuring the absorption peak at 302 nm, with the final concentration calculated using the formula C (mM) = *λ*_Abs_/1.67.

**1.4 ROS detection**

DHU-NO3 (5 μM) and HOCl (15 μM) were mixed in 10 mM PBS buffer (pH 7.4), then 100 μM ABDA was added. The mixtures were irradiated with 658 nm light (20 mW/cm^2^). For dynamic monitoring of ^1^O_2_ production, the mixtures were irradiated for different times, and absorption spectra of ABDA were acquired. ABDA solely and DHU-NO3 (5 μM) incubated with ABDA without HOCl were subjected to irradiation as a control.

**1.5 Detection of NO Release Using the DAN Probe**

Light-triggered NO release was verified using the commercial NO probe DAN, which reacts specifically with NO in the presence of oxygen to yield the strongly fluorescent triazole derivative NAT. DHU-NO3 (5 μM) was first incubated with HOCl (15 μM) in PBS for 5 min to ensure complete ROS-responsive activation. Subsequently, the DAN probe (5 μM) was added to the mixture. This solution was then divided into two aliquots: one was kept in the dark as a control, and the other was irradiated with 405 nm (34 mW cm^−2^) light for 1 h as the experimental group. The fluorescence emission spectra of the solutions were collected immediately after the treatments, and the released NO was analyzed based on the fluorescence intensity of NAT (*λ*_ex_ = 360 nm, *λ*_em_ = 370-600 nm).

**1.6 Preparation of Griess reagent**

Griess Reagent A: 0.1% N-(1-naphthyl)-ethylene diamine dihydrochloride.

Griess Reagent B: 1% sulphanilamide in 5% H_3_PO_4_.

During the test, Griess Reagent A and Griess Reagent B were added to the sample solutions in equal amounts, shaken well, incubated in the dark for 10 min, and then the spectral tests were performed.

**1.7 NO Trapping with PTIO**

PTIO was used as a spin capture agent for NO. An aqueous solution of PTIO (10 uM) and DHU-NO3 (30 μM) + HOCl (90 μM) was irradiated with 405 nm light (34 mW cm^−2^) for 20 min. ESR data were then collected to analyze the NO adduct.

**1.8 HPLC Analysis**

Analytical column: Eclipse Plus C18 4.6*100 mm, 3.5 μm; Injection volume: 20 μL. The mobile phase consisted of acetonitrile and ddH_2_O containing 0.1% (v/v) trifluoroacetic acid. The detector was set at 254 nm for data collection and analysis. First, standard solutions of N-H were prepared at 10 μM and analyzed by HPLC to determine retention times and establish calibration curves. To investigate the responsive behavior of DHU-NO3, samples with the following compositions were prepared in PBS (2 mL) and analyzed by HPLC: DHU-NO3 (10 μM); DHU-NO3 (10 μM) + HOCl (30 μM); DHU-NO3 (10 μM) + HOCl (30 μM) followed by 405 nm irradiation (1 h); and DJNO (10 μM) followed by 405 nm irradiation (34 mW cm^−2^, 1 h). All samples were analyzed immediately after the treatments described. The reaction products were identified by comparing their retention times with those of the standard compounds.

**1.9 Cell Culture**

The 143B cells were cultured in high-glucose Dulbecco's Modified Eagle Medium (DMEM), supplemented with 10% fetal bovine serum (FBS) and 1% Penicillin-Streptomycin, at 37°C in a humidified atmosphere of 5% CO_2_.

**1.10 Cellular Uptake and Imaging**

The cellular uptake of the compounds DHU-NO3, DHUOCl-26, and DHUOCl-27 was assessed using both confocal microscopy and flow cytometry. 143B cells were cultured under standard conditions before experimentation. For imaging, cells were seeded in confocal dishes at a density of 1 × 10^4^ cells/mL and allowed to adhere for 12 h. The compounds from 5 mM DMF stock solutions were diluted with DMEM to a final concentration of 10 μM. After removing the original medium, cells were incubated with the probe-containing medium at 37°C for various durations (0, 1, 2, 4, 8, 12 h). Following incubation, the cells were gently washed twice with PBS to remove non-internalized probes. Imaging was performed using a confocal microscope equipped with a 60 × oil-immersion objective (*λ*_ex_ = 640 nm, *λ*_em_ = 650-750 nm).

For analysis of cellular uptake kinetics by flow cytometry, cells were seeded in 6-well plates at a density of 1×10^5^ cells/mL with 2 mL of medium and allowed to adhere for 12 h. After washing with PBS, the cells were incubated with fresh medium containing 10 μM DHU-NO3 for varying durations (0, 1, 2, 4, 8, and 12 h). Untreated cells served as the negative control. At each time point, the cells were washed twice with PBS, detached using trypsin, harvested, and the resulting cell pellets were resuspended in PBS for immediate flow cytometric analysis.

**1.11 Intracellular NO Detection**

Confocal Laser Scanning Microscopy (CLSM) Imaging Analysis: Intracellular NO levels were detected in situ using the NO-specific fluorescent probe DAF-FM DA. This cell-permeable probe diffuses across the plasma membrane and is hydrolyzed by intracellular esterase to form membrane-impermeable DAF-FM, which exhibits weak baseline fluorescence. A significant fluorescence enhancement occurs upon its specific reaction with NO. Cells pretreated with DHU-NO3 (10 μM) were incubated with 5 μM DAF-FM DA in the dark for 30 min. After washing twice with PBS to remove excess probe, the cells were irradiated with a 405 nm LED light source (22 mW cm^−2^) for 20 min and immediately visualized using the CLSM (*λ*_ex_ = 488 nm, *λ*_em_ = 500-530 nm). Cells treated with DJNO (10 μM) under identical conditions served as the control group.

Flow Cytometric Quantification: 143B cells were seeded in 6-well plates at a density of 1×10^5^ cells/mL (2 mL/well) and cultured for 12 h to allow complete adhesion. After co-incubation with 10 μM DHU-NO3 for 12 h, cells were loaded with 5 μM DAF-FM DA in the dark for 25 min. Following PBS washing, the cells were irradiated with a 405 nm LED light source (22 mW cm^−2^) for varying durations (0, 5, 10, 15, 20 min) and then incubated for an additional 20 min. The cells were then detached using trypsin, resuspended in PBS, and immediately analyzed by flow cytometry.

**1.12 Cytotoxicity Assessment**

The cytotoxicity of the compounds was evaluated using the CCK-8 assay. 143B cells were seeded into 96-well plates at a density of 1 × 10^4^ cells per well and cultured for 24 h under standard conditions (37°C, 5% CO_2_). For dark cytotoxicity assessment, cells were treated with various concentrations (0, 5, 10, 25, and 50 μM) of DHU-NO3, DHUOCl-26, DHUOCl-27, or DJNO for 24 h. For phototoxicity evaluation, cells were incubated with the compounds for 12 h followed by specific light irradiation protocols: DHUOCl-26 and DHUOCl-27 groups received 650 nm laser irradiation (14 mW cm^−2^, 5 min); DJNO group was exposed to 405 nm LED light (22 mW cm^−2^, 20 min); and the DHU-NO3 group was divided into two subgroups receiving either 405 nm irradiation alone or combined 405 nm and 650 nm irradiation. After their respective treatments, all cells were incubated for an additional 12 h. Subsequently, 100 μL of DMEM containing 10% CCK-8 reagent was added to each well, and the mixture was incubated for 2 h at 37°C. The absorbance at 450 nm was measured using a microplate reader to determine cell viability.

**1.13 Cell Viability Staining Assay**

Cell viability was assessed using a calcein-AM/PI double-staining kit following various treatment conditions. 143B cells were cultured on 35 mm glass-bottom dishes for 24 h at 37°C before experimentation. The cells were divided into four treatment groups: irradiation with 405 nm (22 mW cm^−2^) light for 20 min; irradiation with 405 nm (20 min) followed by 650 nm (14 mW cm^−2^, 5 min); incubation with DHU-NO3 (10, 25, or 50 μM) for 12 h followed by 405 nm irradiation (20 min); incubation with DHU-NO3 (10, 25, or 50 μM) for 12 h followed by sequential irradiation with 405 nm (20 min) and 650 nm (5 min). After their respective treatments, all cells were stained with calcein-AM and PI solutions for 30 min at 37°C according to the manufacturer’s protocol. Following three washes with PBS, the samples were immediately examined under CLSM. The imaging parameters were set as follows: for the green channel (calcein-AM, *λ*_ex_ = 488 nm, *λ*_em_ = 500-545 nm); for the red channel (PI, *λ*_ex_ = 535 nm, *λ*_em_ = 600-630 nm).

**1.14 Detection of Intracellular ROS Generation**

Intracellular ROS levels upon light irradiation were monitored using the fluorescent probe DCFH-DA. This cell-permeable probe is hydrolyzed by intracellular esterase to non-fluorescent DCFH, which is subsequently oxidized by ROS to form highly fluorescent 2’, 7’-dichlorofluorescein (DCF), with fluorescence intensity correlating with intracellular ROS levels. 143B cells were incubated with 10 μM of DHU-NO3, DHUOCl-26, DHUOCl-27, or DJNO for 12 h, followed by treatment with 10 μM DCFH-DA for 30 min. After washing with PBS, the cells were subjected to specific light irradiation protocols: the DHUOCl-26 and DHUOCl-27 groups received 650 nm (14 mW cm^−2^) laser irradiation for 5 min; the DJNO group was exposed to 405 nm (22 mW cm^−2^) LED light for 20 min; and the DHU-NO3 group was treated either with 405 nm light alone or with sequential irradiation (405 nm for 20 min followed by 650 nm for 5 min). CLSM was subsequently performed to evaluate ROS generation levels before and after irradiation across all experimental groups (*λ*_ex_ = 488 nm, *λ*_em_ = 500-550 nm).

**1.15 ATP Content Measurement**

Intracellular ATP levels were quantified using a commercial ATP assay kit. 143B cells were divided into ten experimental groups: Control, DHUOCl-26, DHUOCl-26 + 650 nm (14 mW cm^−2^, 5 min), DHUOCl-27, DHUOCl-27 + 650 nm, DJNO, DJNO + 405 nm (22 mW cm^−2^, 20 min), DHU-NO3, DHU-NO3 + 405 nm, and DHU-NO3 + 405 nm + 650 nm. Following the respective treatments, the culture medium was carefully removed. Cells were then lysed by adding the provided lysis buffer. The resulting lysates were centrifuged at 12000 × g for 5 min at 4°C to pellet cellular debris. The supernatants were collected and detected by the Enhanced ATP Assay Kit.

**1.16 Intracellular GSH Depletion Assay**

Cellular GSH levels were evaluated using a commercial GSH assay kit. 143B cells were divided into four experimental groups: Control, DHU-NO3, DHU-NO3 + 405 nm (22 mW cm^−2^, 20 min) irradiation, and DHU-NO3 + 405 nm + 650 nm (14 mW cm^−2^, 5 min) irradiation. Following the respective treatments, cells were washed with PBS and subsequently lysed according to the kit’s instructions. The resulting lysates were processed and analyzed according to the manufacturer’s protocol to determine intracellular GSH levels.

**1.17 Western Blot Analysis**

The protein expression levels of Cleaved PARP, Cleaved Caspase-3, CRT, and c-MYC were analyzed by western blotting. 143B cells were divided into different experimental groups according to the respective treatments. After treatment completion, cells were lysed using RIPA buffer. The protein concentration of each lysate was determined using a BCA protein assay kit. Equal amounts of protein from different treatment groups were separated by SDS-PAGE electrophoresis and subsequently transferred to methanol-activated PVDF membranes. Electrophoresis was performed at 100 V until the bromophenol blue dye reached the bottom of the gel. Following transfer, the membranes were blocked with 5% skim milk for 1 h at room temperature. The membranes were incubated overnight at 4°C with specific primary antibodies, followed by 1 h at room temperature with corresponding secondary antibodies. After each antibody incubation, the membranes were washed three times with TBST. Protein bands were visualized with a gel imaging system, and densitometry was performed with ImageJ.

**1.18 In vivo fluorescence imaging**

Our clinical sample study was approved by the Institutional Research Ethics Committee of Shanghai General Hospital, Shanghai Jiao Tong University School of Medicine (Approval No. 2021KY103). All procedures for consideration of animal welfare were reviewed and approved by the Animal Care and Use Committee of the Shanghai General Hospital (2025AW055). Male BALB/c nude mice (4-6 weeks) were purchased from Shanghai Ji Hui Laboratory Animal Care Co., Ltd. The subcutaneous tumor model was established by injecting 100 μL of 2 × 10^6^ 143B cells suspended in PBS into the subcutaneous tissue. In vivo fluorescence imaging was performed using an IVIS Lumina Series III small-animal imaging system with a 660 nm excitation laser and a 710 ± 20 nm collection filter. Sequential images were acquired at predetermined time points post-injection to monitor the prodrug's distribution and activation.

**1.19 In Vivo Therapeutic Efficacy Evaluation**

The in vivo antitumor efficacy was evaluated in 143B osteosarcoma xenograft model. When tumor volumes reached approximately 100-120 mm^3^, tumor-bearing mice were randomly allocated into seven experimental groups (n = 6 per group). To each group, 50 μL of prodrug or reference compounds were intratumorally injected. 1: Control (no treatment); 2: MB; 3: DHU-NO3; 4: DJNO + 405 nm (180 mW cm^−2^, 10 min); MB + 650 nm (300 mW cm^−2^, 5 min); DHU-NO3 + 405 nm (180 mW cm^−2^, 10 min); DHU-NO3 + 405 nm (10 min) + 650 nm (5 min). Tumor volumes and body weight were recorded for 14 days after various administrations. Tumor volume (V) was calculated using the formula: *V* = (*length* × *width*^2^)/2. Upon completion of the therapeutic study, all animals were euthanized. Tumors and major organs (heart, liver, spleen, lung, and kidneys) were harvested for subsequent pathological examination. Tissue sections underwent comprehensive histological analysis, including hematoxylin and eosin (H&E) staining, TUNEL apoptosis assay, and Ki-67 immunohistochemistry, according to standardized protocols.

**1.20 Statistical Analysis**

Data were expressed as mean ± standard deviation (SD). Data were analyzed by GraphPad Prism 10 and Origin 2024. Unpaired Student’s t tests (two groups) or one-way ANOVA with Tukey’s post hoc test (n ≥ 3) were used for comparisons. Significant differences of p < 0.05, p < 0.01, p < 0.001, and p < 0.0001 were marked with *, **, ***, and ****, respectively. Non-significant differences are labeled as “ns” (not significant).

**2 Supplemental Figures**


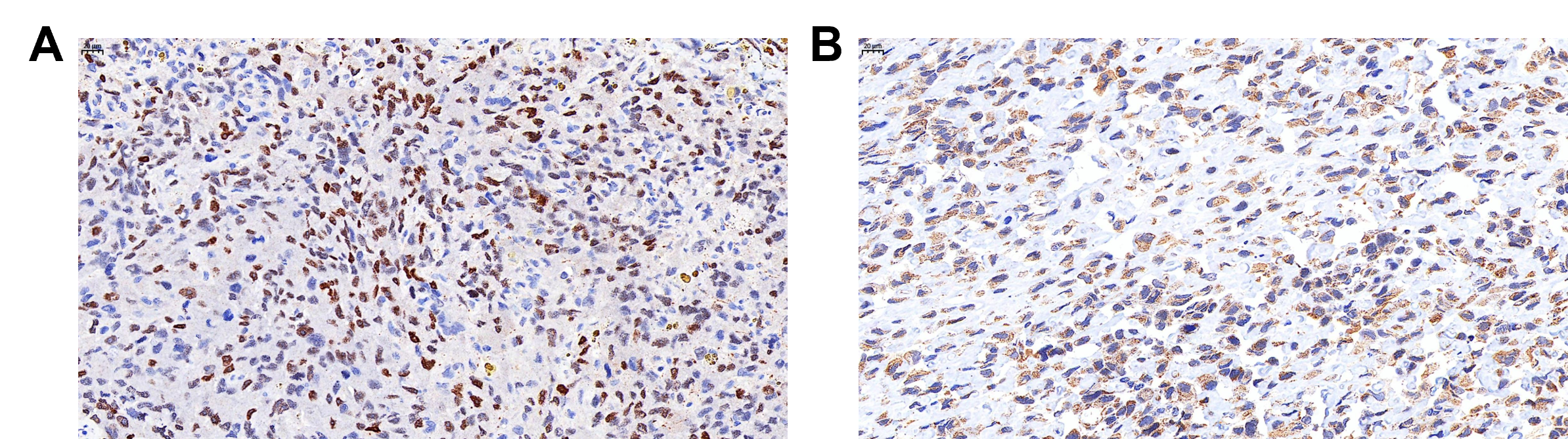


**Figure S1.** Representative immunohistochemical staining images of c-MYC protein in (A) human osteosarcoma clinical samples and (B) patient-derived xenograft (PDX) tumor tissues.


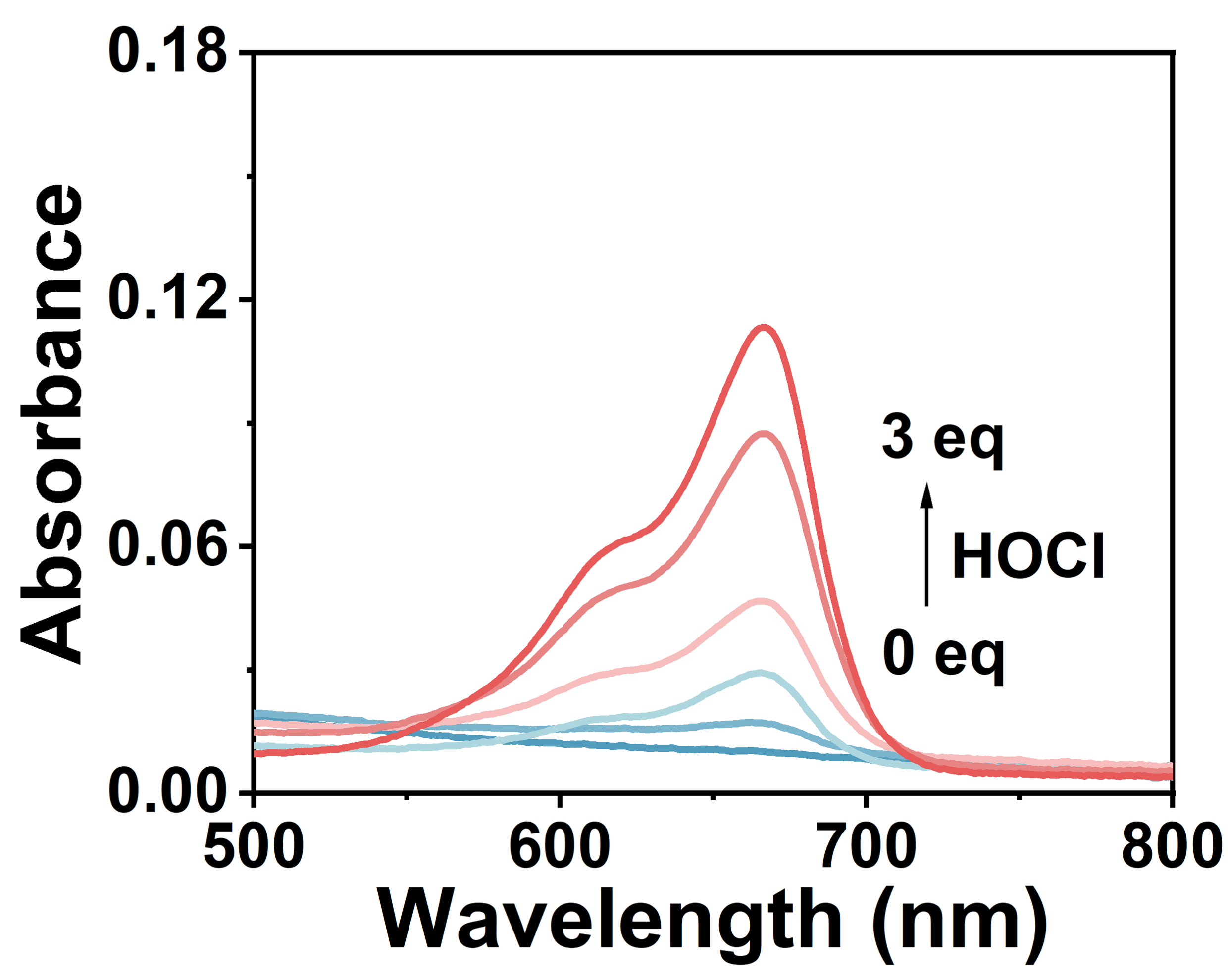


**Figure S2.** Absorption spectra of DHU-NO3 (5 μM) upon addition of varying concentrations of HOCl (0-3 eq) in PBS.


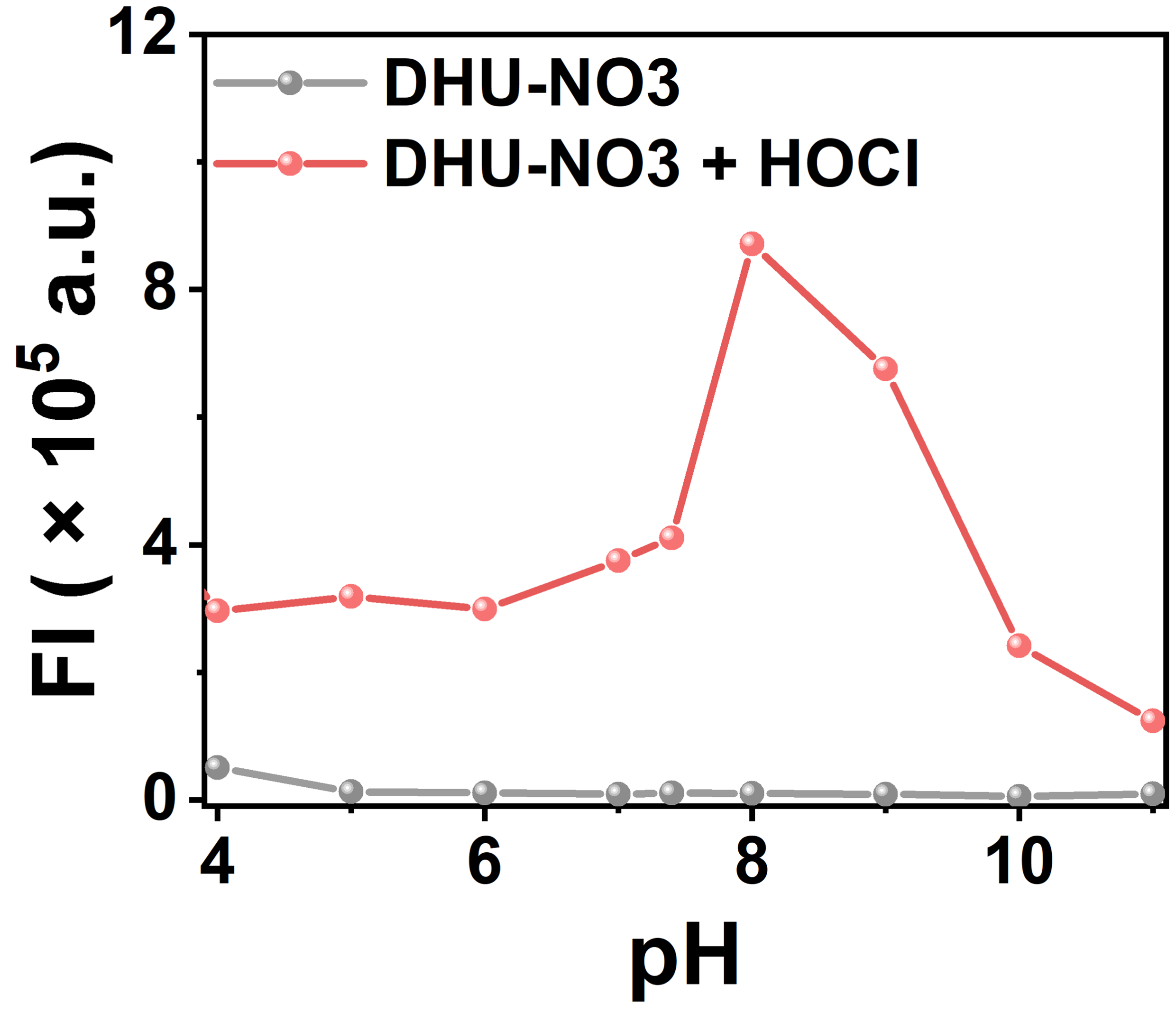


**Figure S3.** Fluorescence intensity of DHU-NO3 (5 μM) at 686 nm after reaction with 15 μM HOCl under different pH conditions.


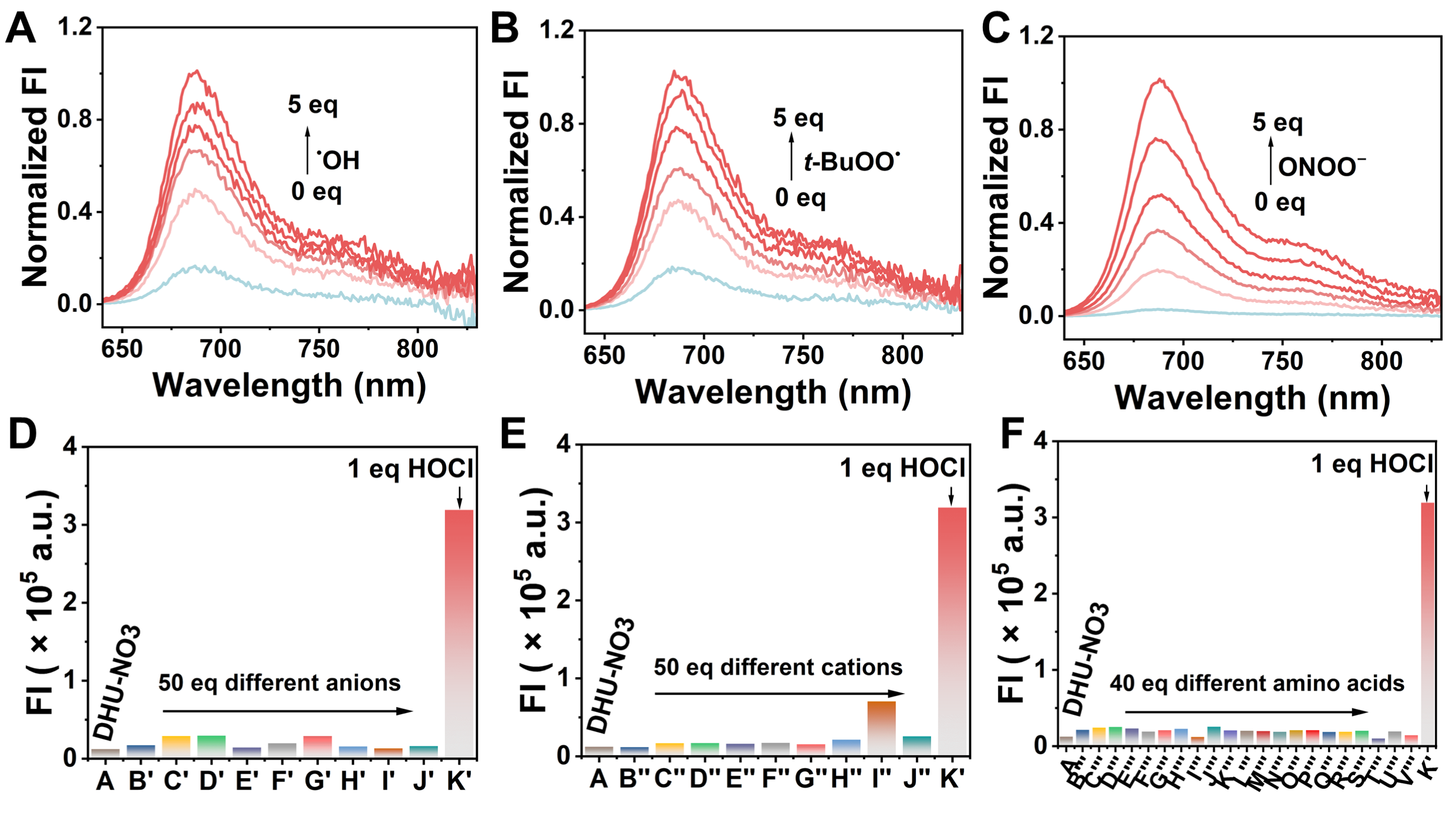


**Figure S4.** A-C) Fluorescence emission spectra (*λ*_ex_ = 620 nm) of DHU-NO3 (10 μM) in the presence of increasing concentrations of ^•^OH (A), *t*-BuOO^•^ (B), and ONOO^−^ (C). D) Fluorescence intensity at 686 nm after treatment with different anions (A: DHU-NO3 only; B’-J’: CH_3_COO^−^, CO_3_^2−^, SO_4_^2−^, F^−^, Cl^−^, I^−^, NO_2_^−^, S_2_O_3_^2−^, ClO_4_^−^; K': HOCl); E) Fluorescence intensity at 686 nm after treatment with different cations (A: DHU-NO3 only; B''-J’’: NH_4_^+^, Na^+^, Mg^2+^, Al^3+^, K^+^, Ca^2+^, Fe^3+^, Cu^2+^, Ni^2+^; K': HOCl); F) Fluorescence intensity at 686 nm after treatment with different amino acids and GSH (A: DHU-NO3 only; B'''-V''': Leu, Pro, Gly, Gln, Glu, Met, Lys, Trp, Ser, Thr, Asp, Ile, Val, His, Ala, Cys, Phe, Asn, Tyr, Arg, GSH; K': HOCl).


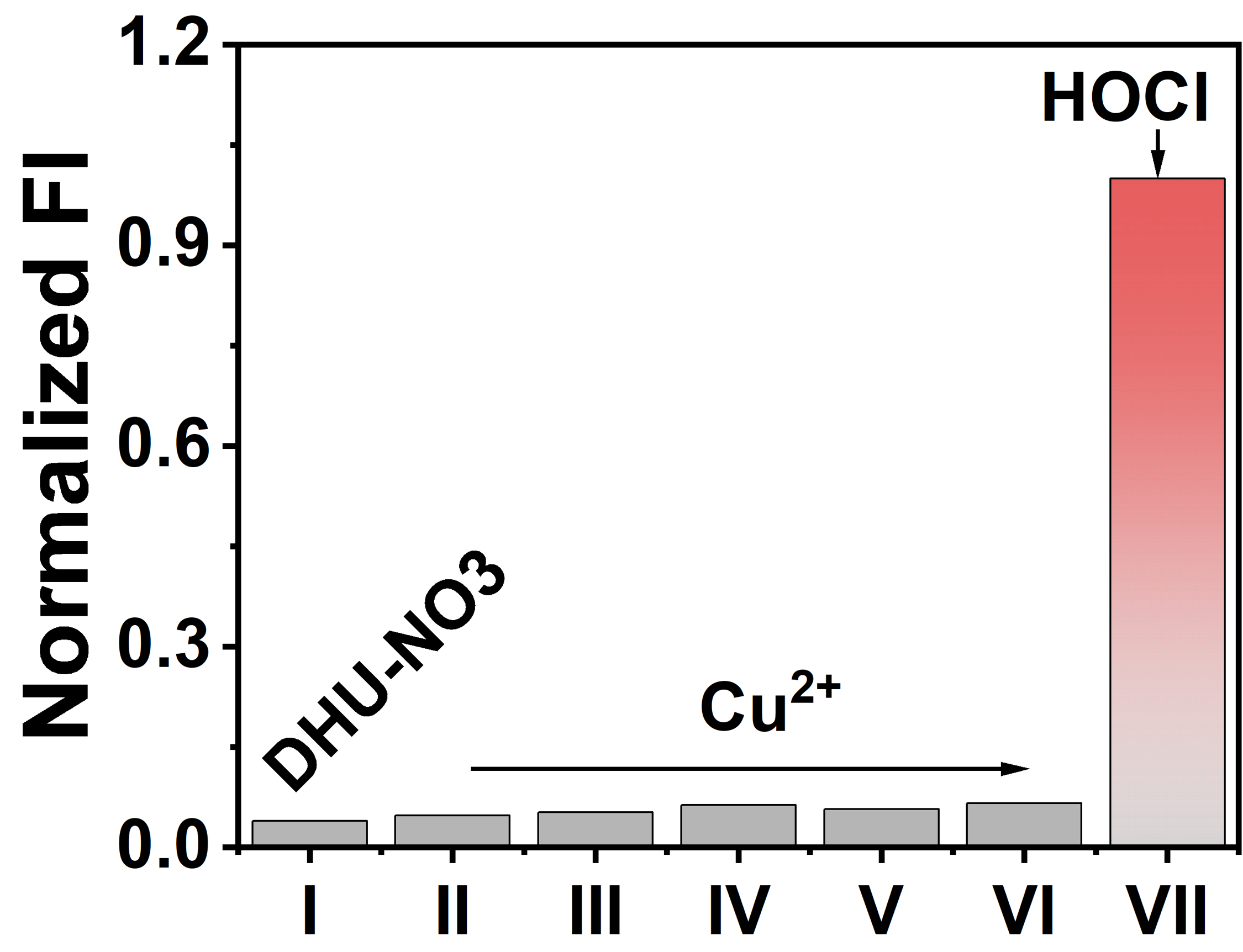


**Figure S5.** Fluorescence intensity at 686 nm of DHU‑NO3 (5 μM) after treatment with increasing concentrations of Cu^2+^. I: DHU‑NO3 only; II-VI: DHU‑NO3 with Cu^2+^ at 3, 5, 10, 15, and 20 μM, respectively; VII: DHU‑NO3 with HOCl (3 eq) as a positive control.


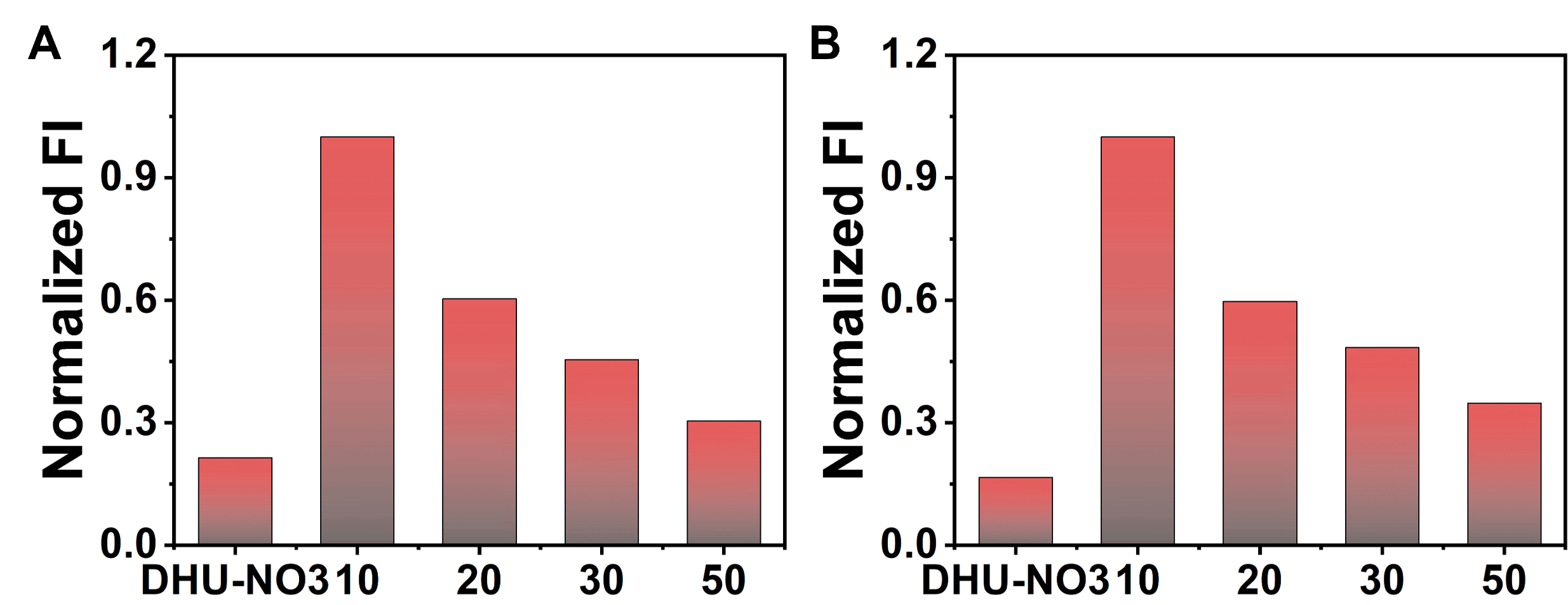


**Figure S6.** Fluorescence intensity at 686 nm of DHU‑NO3 (5 μM) upon addition of 3 eq of HOCl in the presence of increasing concentrations of (A) GSH and (B) Cys.


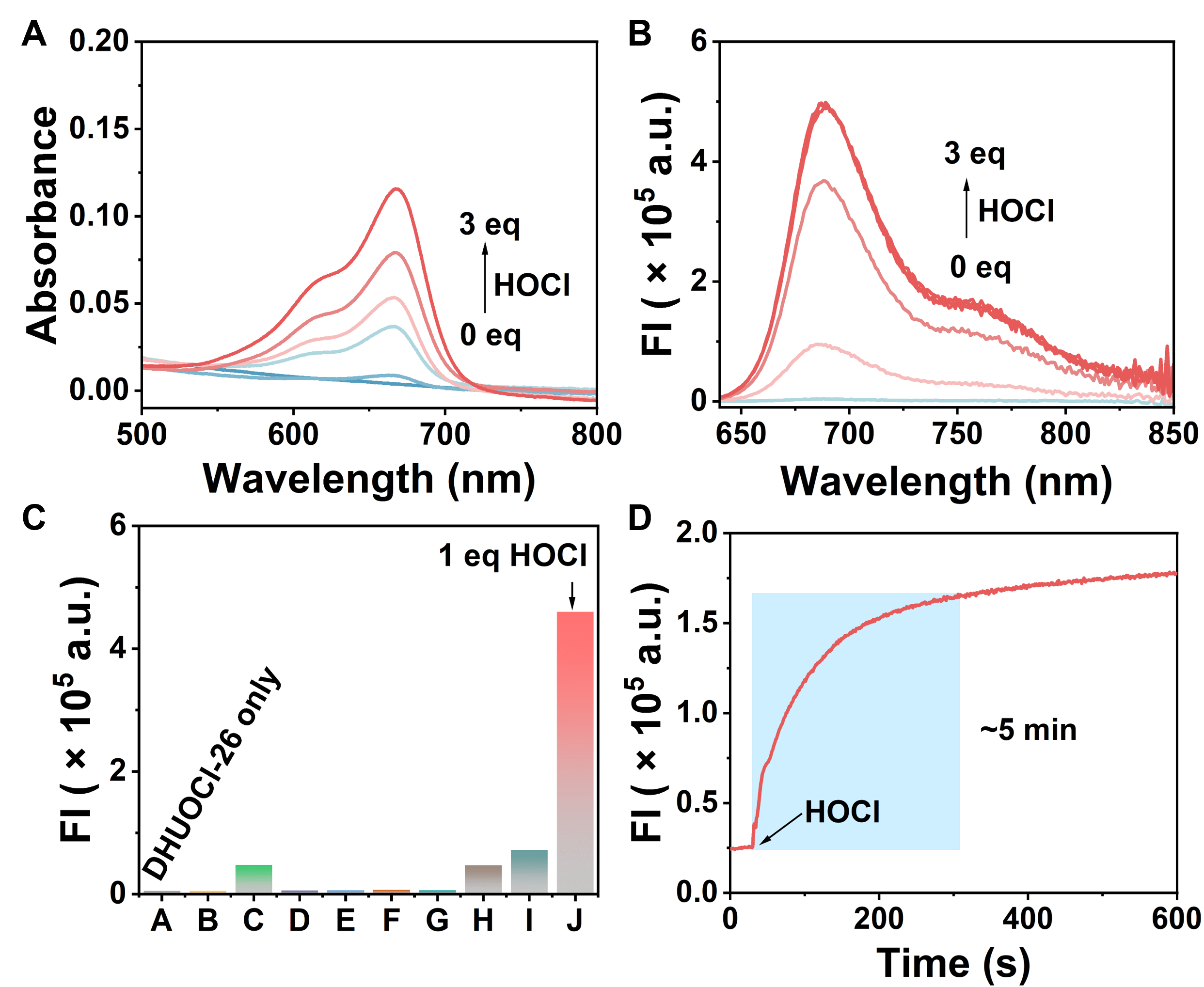


**Figure S7.** A) Absorption spectra of DHUOCl-26 (5 μM) upon addition of varying concentrations of HOCl (0-3 eq) in PBS. B) Fluorescence emission spectrum of DHUOCl-26 (5 μM) upon addition of varying concentrations of HOCl (0-3 eq) in PBS (*λ*_ex_ = 620 nm). C) Fluorescence intensity at 686 nm after DHUOCl-26 (5 μM) treatment with various ROS (A: DHUOCl-26 only, B-I: H_2_O_2_, ^•^OH, TBHP, ROO^•^, NO, O_2_^−^, t-BuOO^•^, ONOO^−^, J: HOCl). D) Time-dependent fluorescence intensity change of DHUOCl-26 (5 μM) at 686 nm after HOCl addition (3 eq, indicated by black arrow).


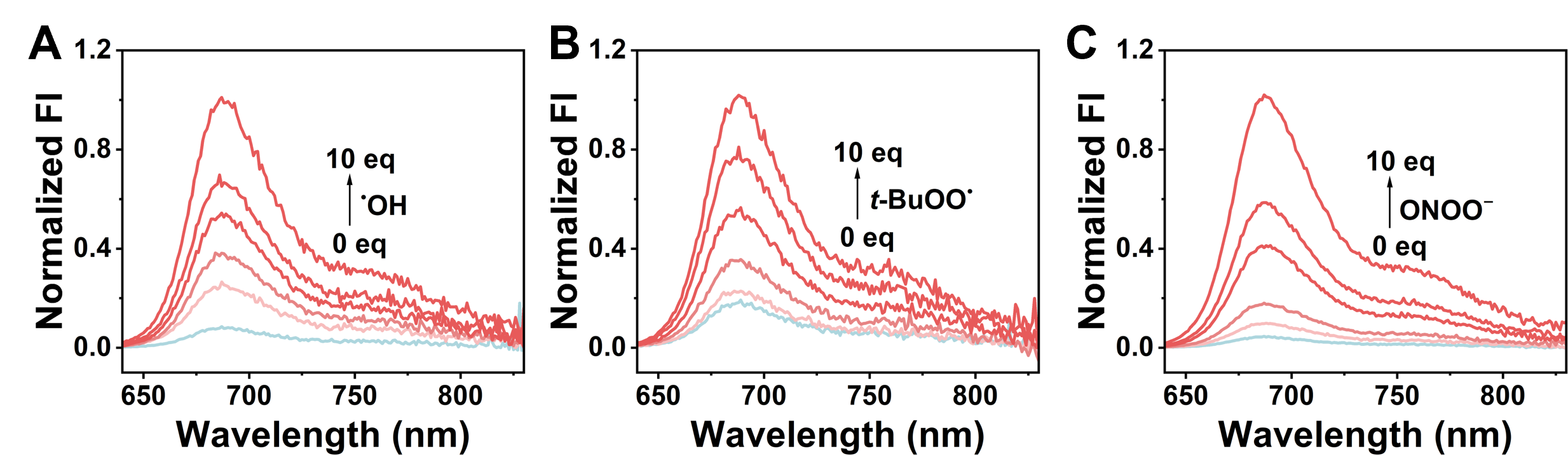


**Figure S8.** Fluorescence emission spectra (*λ*_ex_ = 620 nm) of DHUOCl-26 (5 μM) in the presence of increasing concentrations of ^•^OH (A), *t*-BuOO^•^ (B), and ONOO^−^ (C).


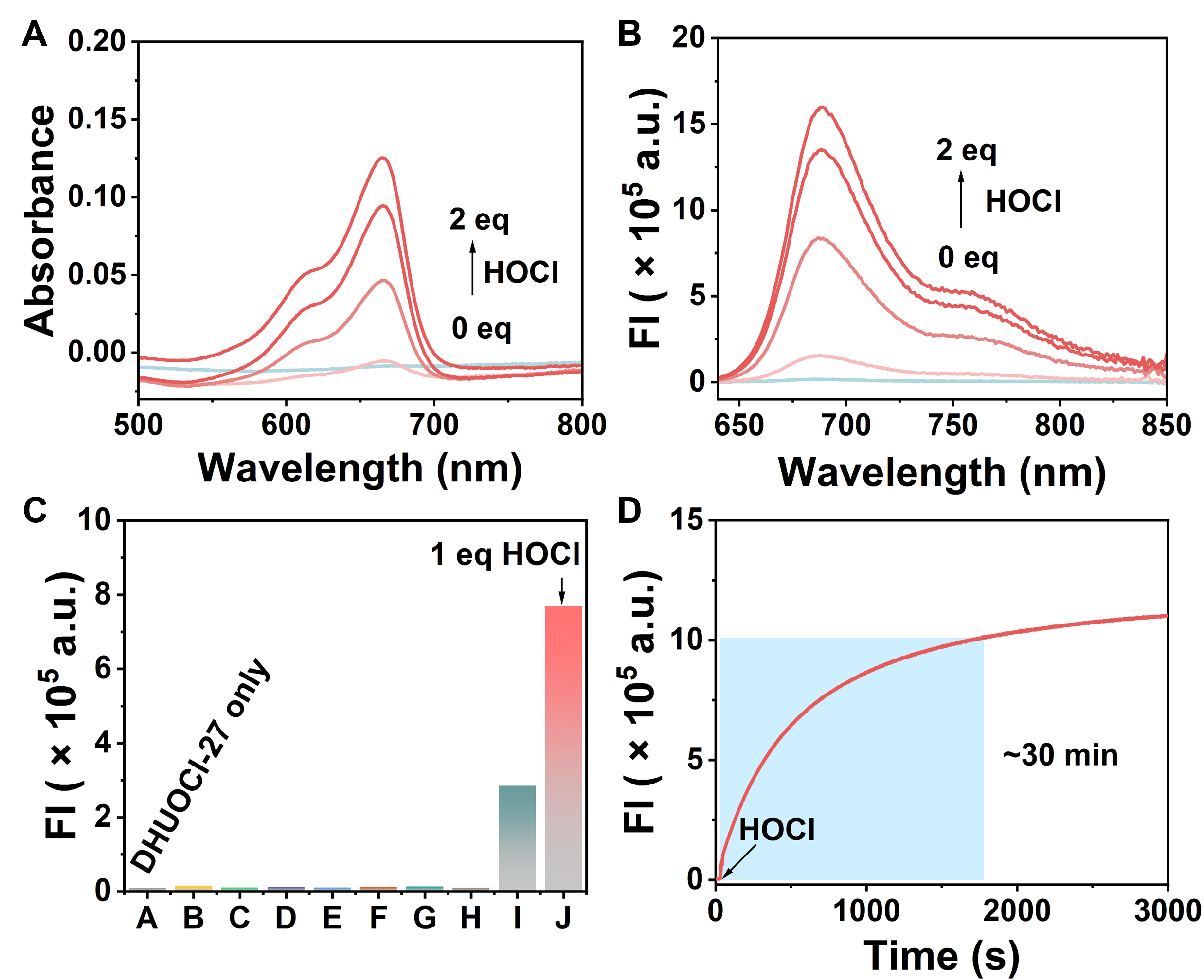


**Figure S9.** Spectral characterization of DHUOCl-27. A) Absorption spectra of DHUOCl-27 (5 μM) upon addition of varying concentrations of HOCl (0-2 eq) in PBS. B) Fluorescence emission spectrum of DHUOCl-27 (5 μM) upon addition of varying concentrations of HOCl (0-2 eq) in PBS (*λ*_ex_ = 620 nm). C) Fluorescence intensity at 686 nm after DHUOCl-27(5 μM) treatment with various ROS (A: DHUOCl-27 only, B-I: H_2_O_2_, ^•^OH, TBHP, ROO^•^, NO, O_2_^−^, t-BuOO^•^, ONOO^−^, J: HOCl). D) Time-dependent fluorescence intensity change of DHUOCl-27 (5 μM) at 686 nm after HOCl addition (2 eq, indicated by black arrow).


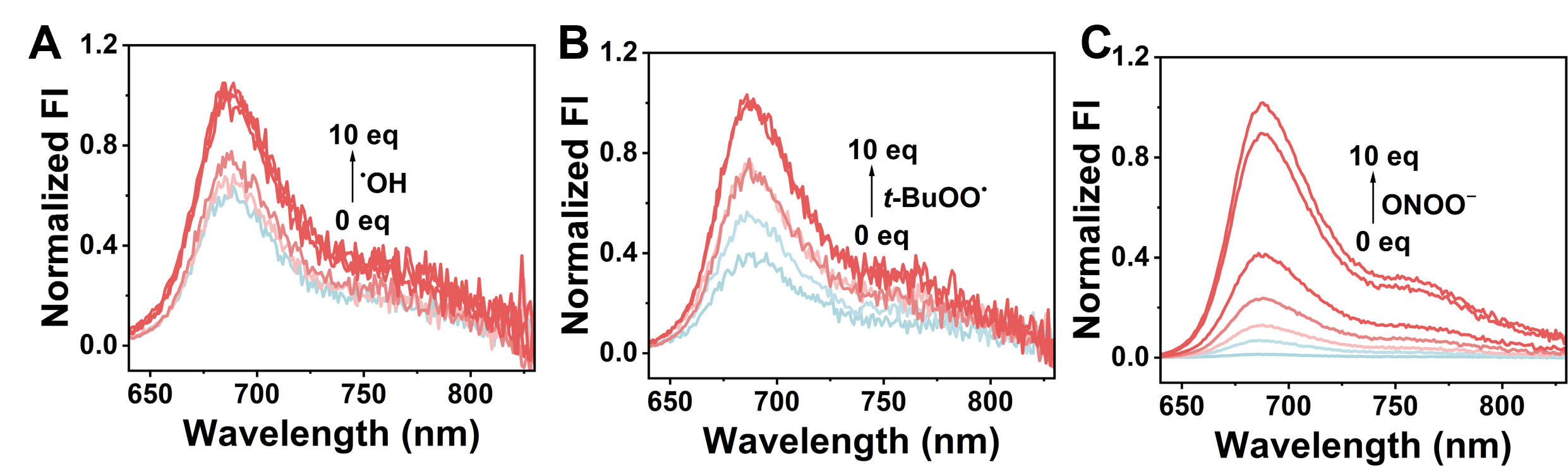


**Figure S10.** Fluorescence emission spectra (*λ*_ex_ = 620 nm) of DHUOCl-27 (5 μM) in the presence of increasing concentrations of ^•^OH (A), *t*-BuOO^•^ (B), and ONOO^−^ (C).


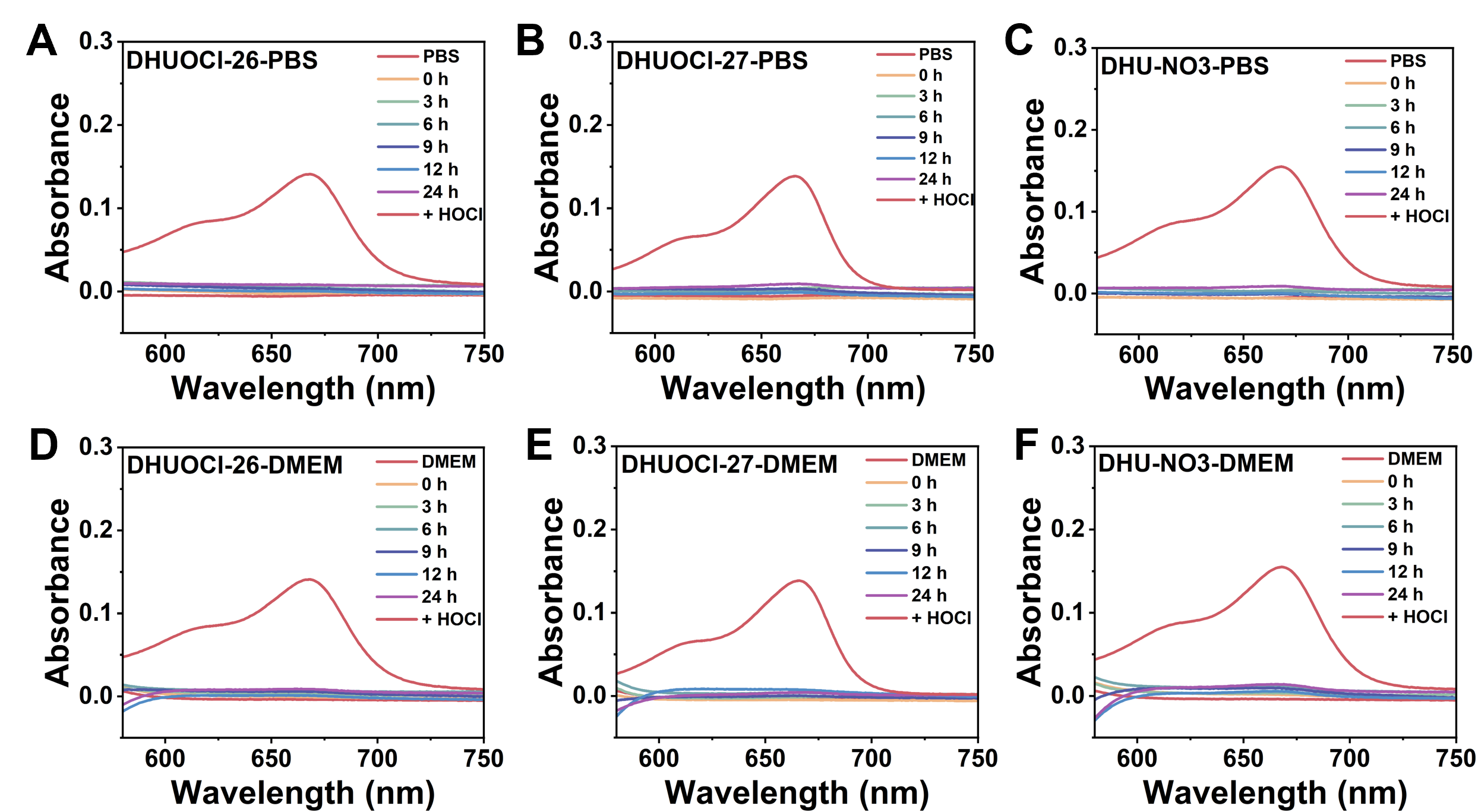


**Figure S11.** UV‑vis absorption spectra of DHU‑NO3, DHUOCl‑26, and DHUOCl‑27 in PBS (A-C) and in DMEM containing 10% FBS (D-F) recorded at 0, 3, 6, 9, 12, 24 h and after HOCl addition.


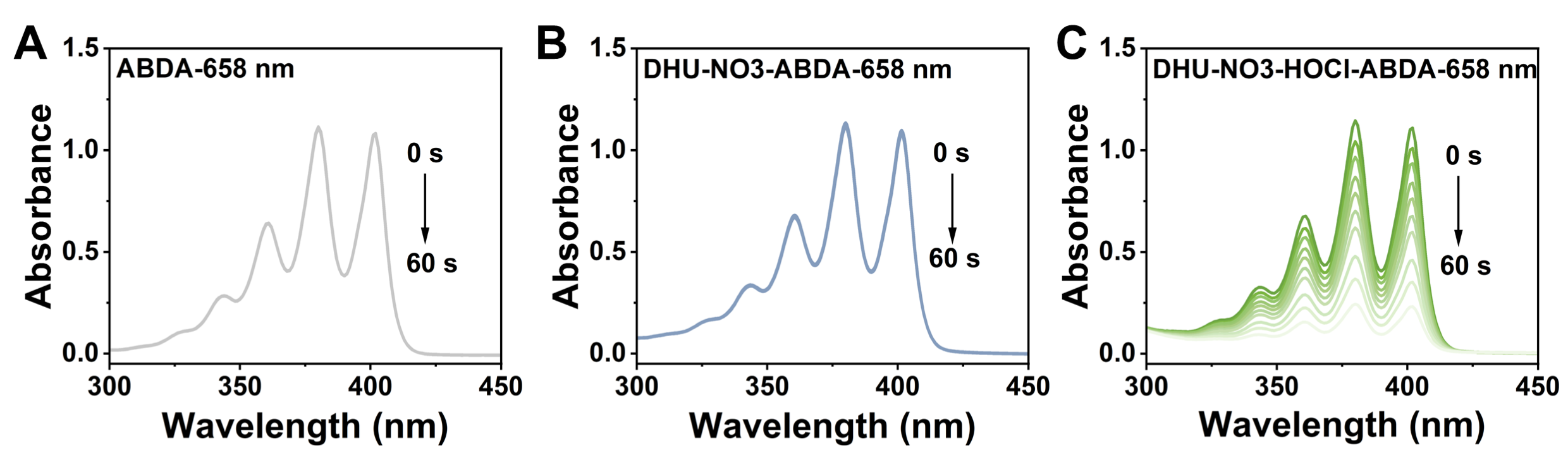


**Figure S12.** Absorption spectra of ABDA (100 μM) under 658 nm laser irradiation (20 mW/cm⁻^2^) in the presence of (A) ABDA alone, (B) DHU-NO3 (5 μM) + ABDA, or (C) DHU-NO3 (5 μM) pretreated with HOCl (3 eq) + ABDA.


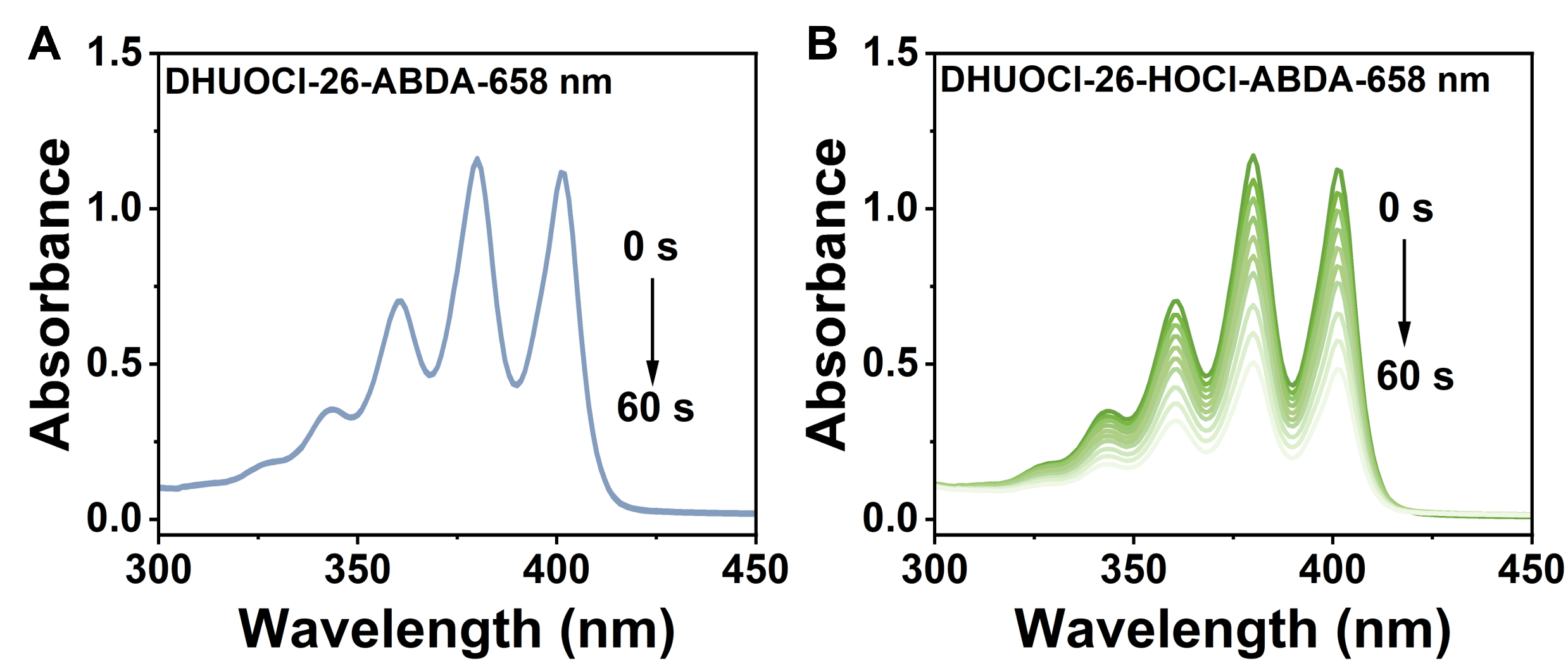


**Figure S13.** Absorption spectra of ABDA (100 μM) under 658 nm laser irradiation (20 mW/cm⁻^2^) in the presence of (A) DHUOCl-26 (5 μM) + ABDA, or (C) DHUOCl-26 (5 μM) pretreated with HOCl (3 eq) + ABDA.


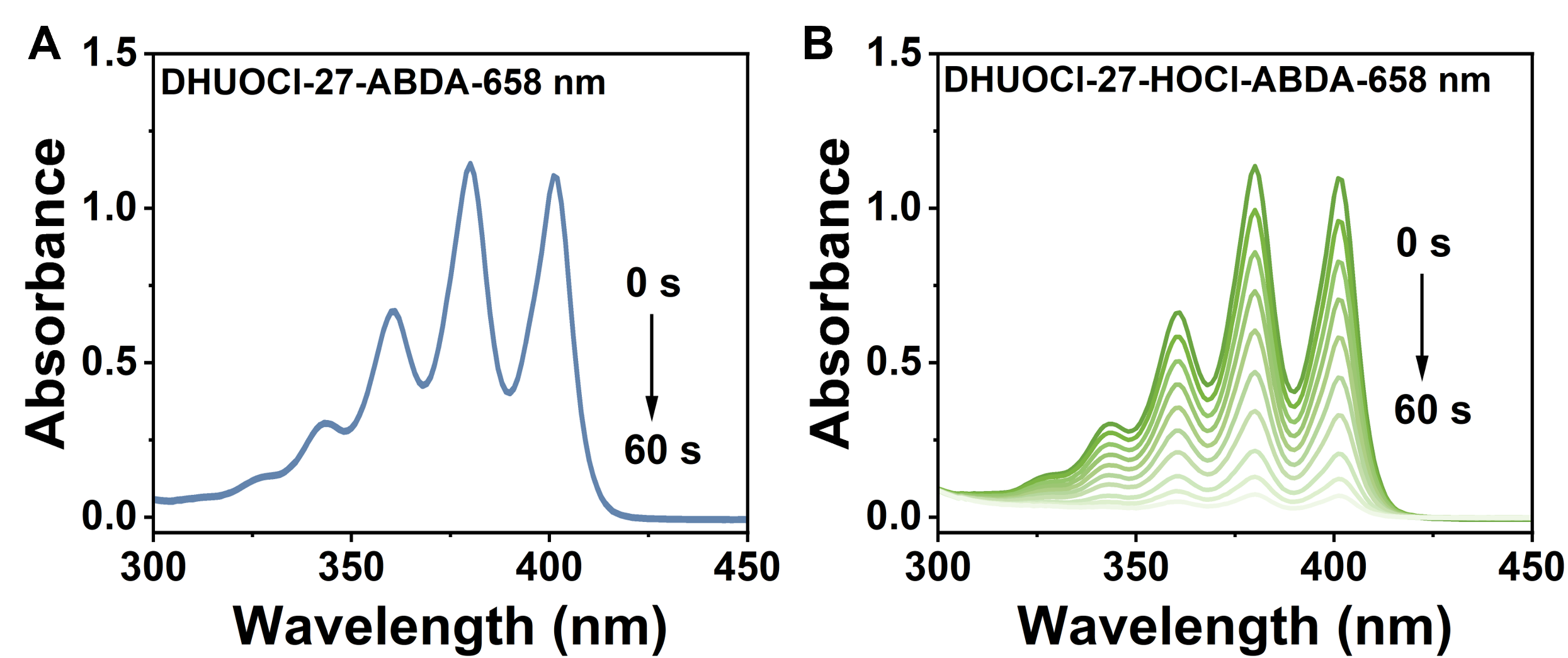


**Figure S14.** Absorption spectra of ABDA (100 μM) under 658 nm laser irradiation (20 mW/cm⁻^2^) in the presence of (A) DHUOCl-27 (5 μM) + ABDA, or (C) DHUOCl-27 (5 μM) pretreated with HOCl (2 eq) + ABDA.


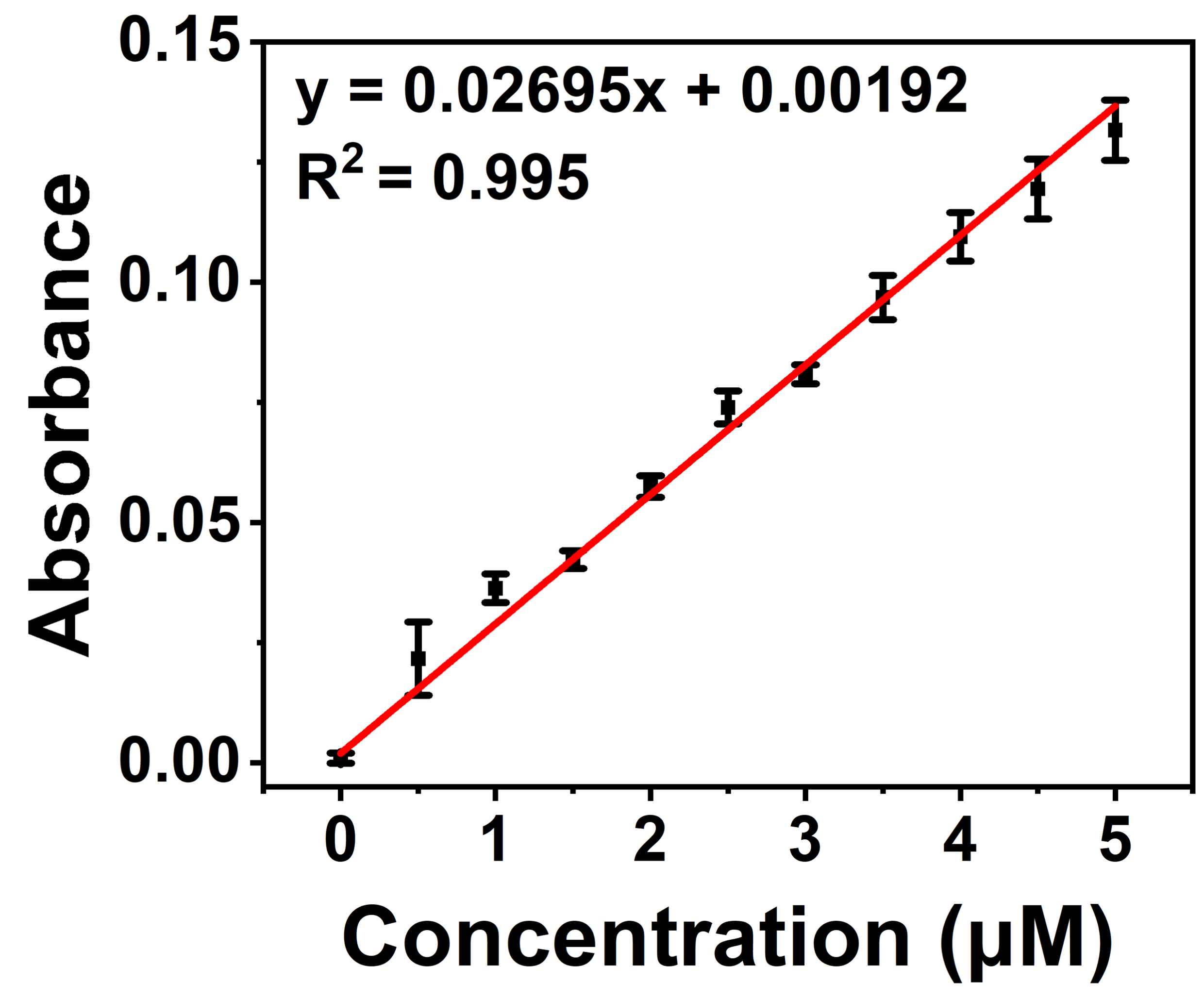


**Figure S15.** Standard curve for NO quantification. Griess reagent assay was performed with sodium nitrite standards (0-5 μM) to establish the calibration for NO measurement.


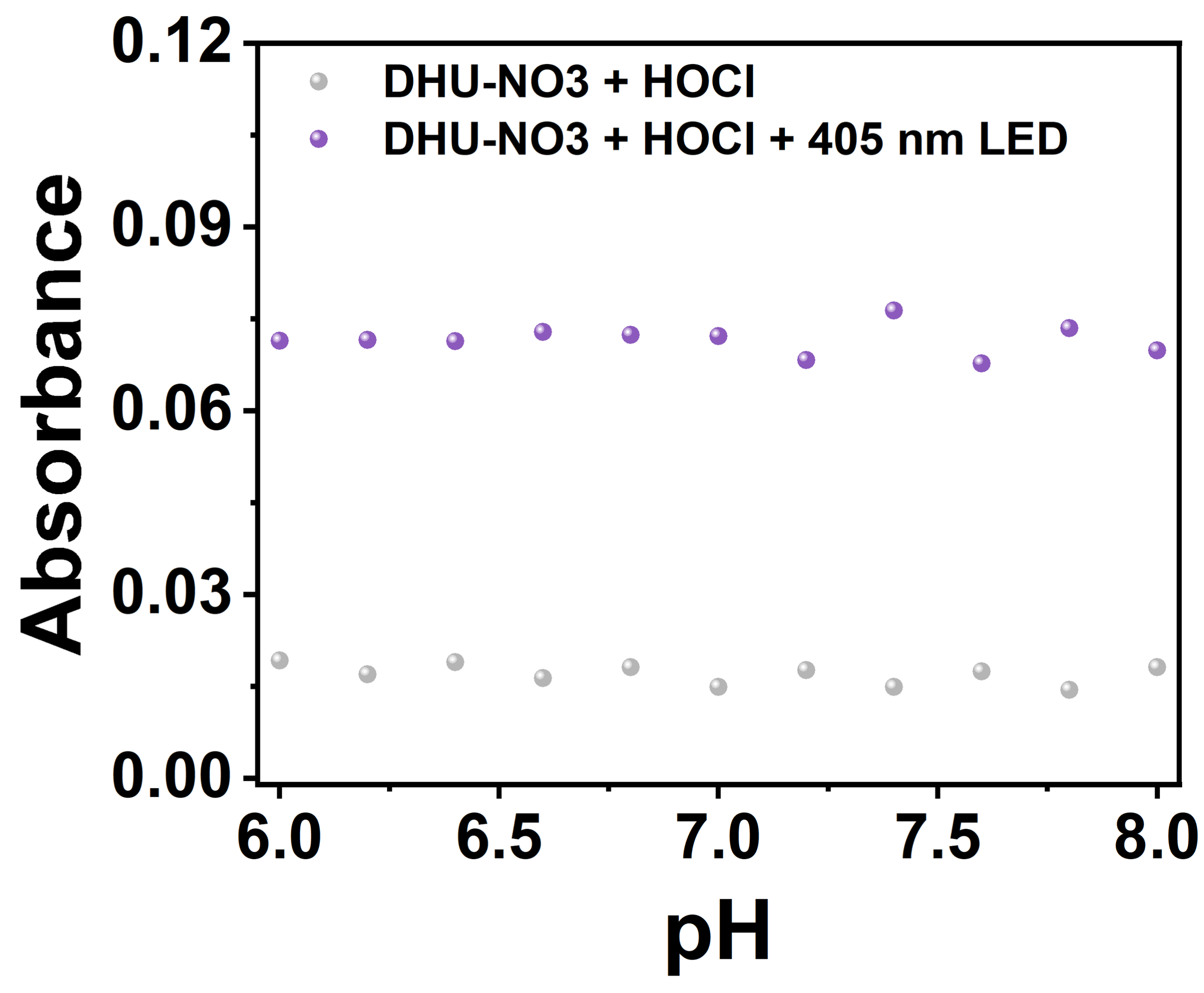


**Figure S16.** pH-dependent NO release from DHU‑NO3. DHU‑NO3 (5 μM) was pretreated with HOCl (15 μM) in PBS at different pH values, followed by 405 nm LED irradiation (34 mW cm^−2^, 1 h).


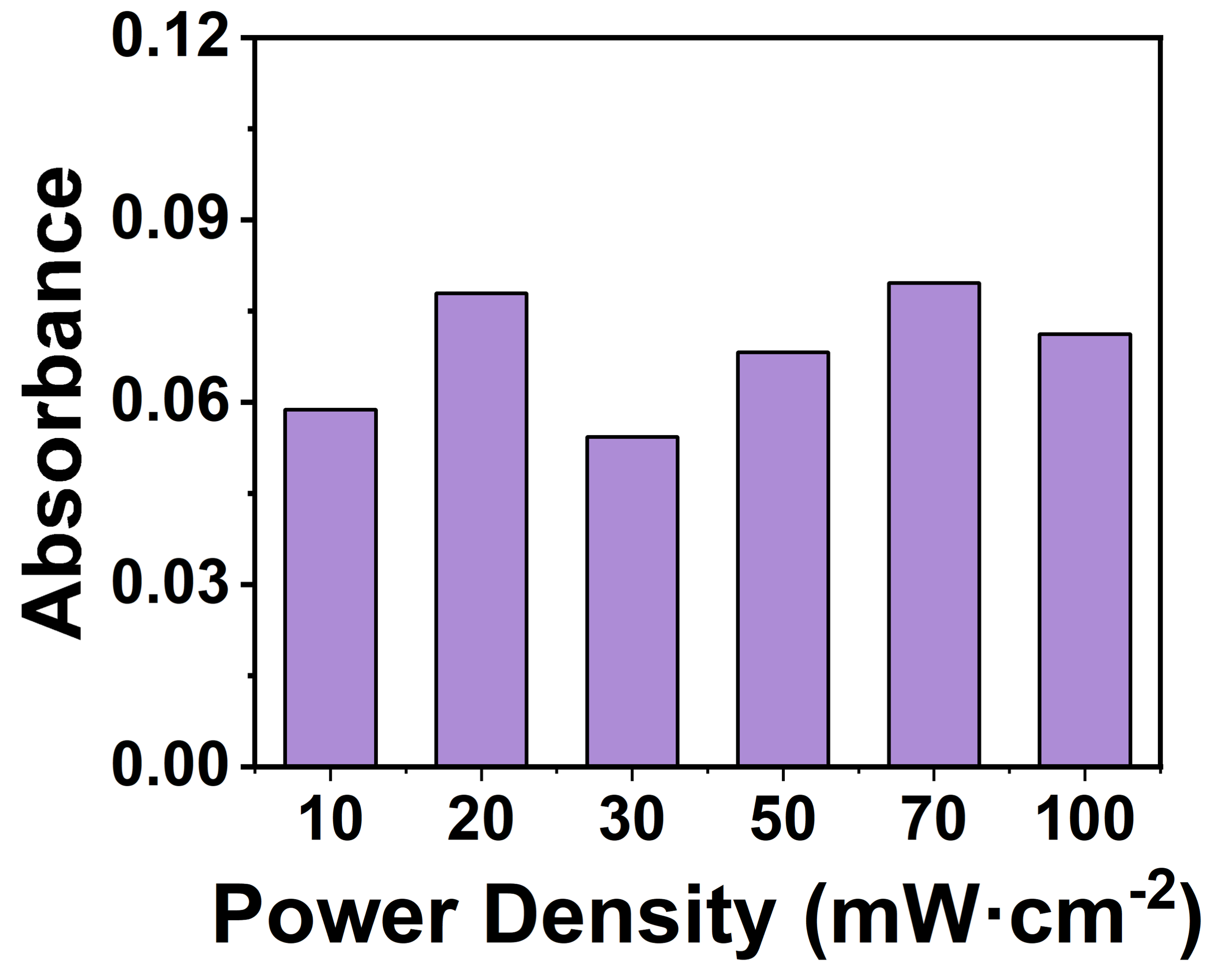


**Figure S17.** Power density-dependent NO release from DHU-NO3. NO release from DHU-NO3 and HOCl in PBS under 405 nm (1h) LED irradiation at different power densities.


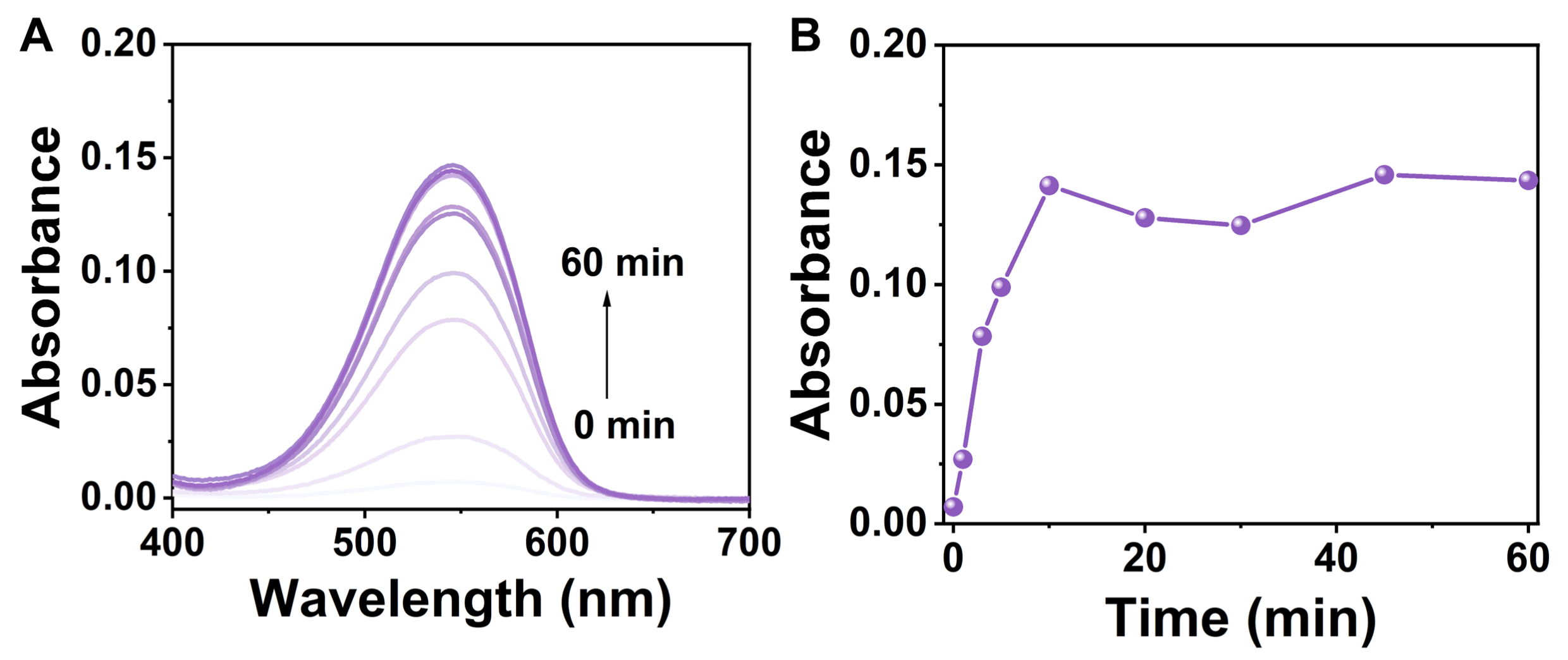


**Figure S18.** Photocontrolled NO release kinetics of the donor DJNO. (A) Absorption spectral changes of DJNO (5 μM) after 405 nm (34 mW cm^−2^) LED illumination for different durations. (B) Quantitative analysis of absorption changes at 550 nm.


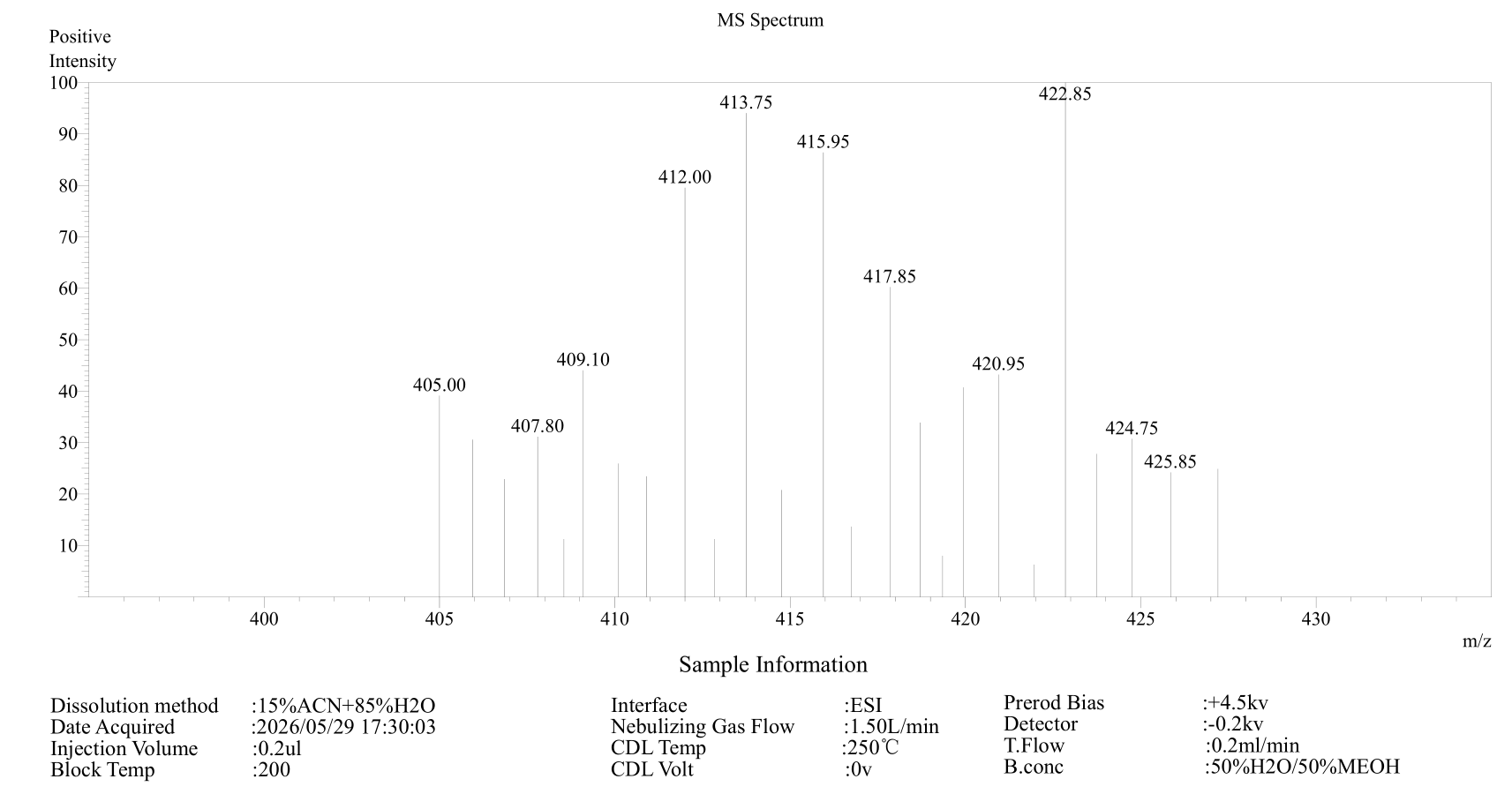


**Figure S19.** MS spectrum of the QM‑GSH adduct.


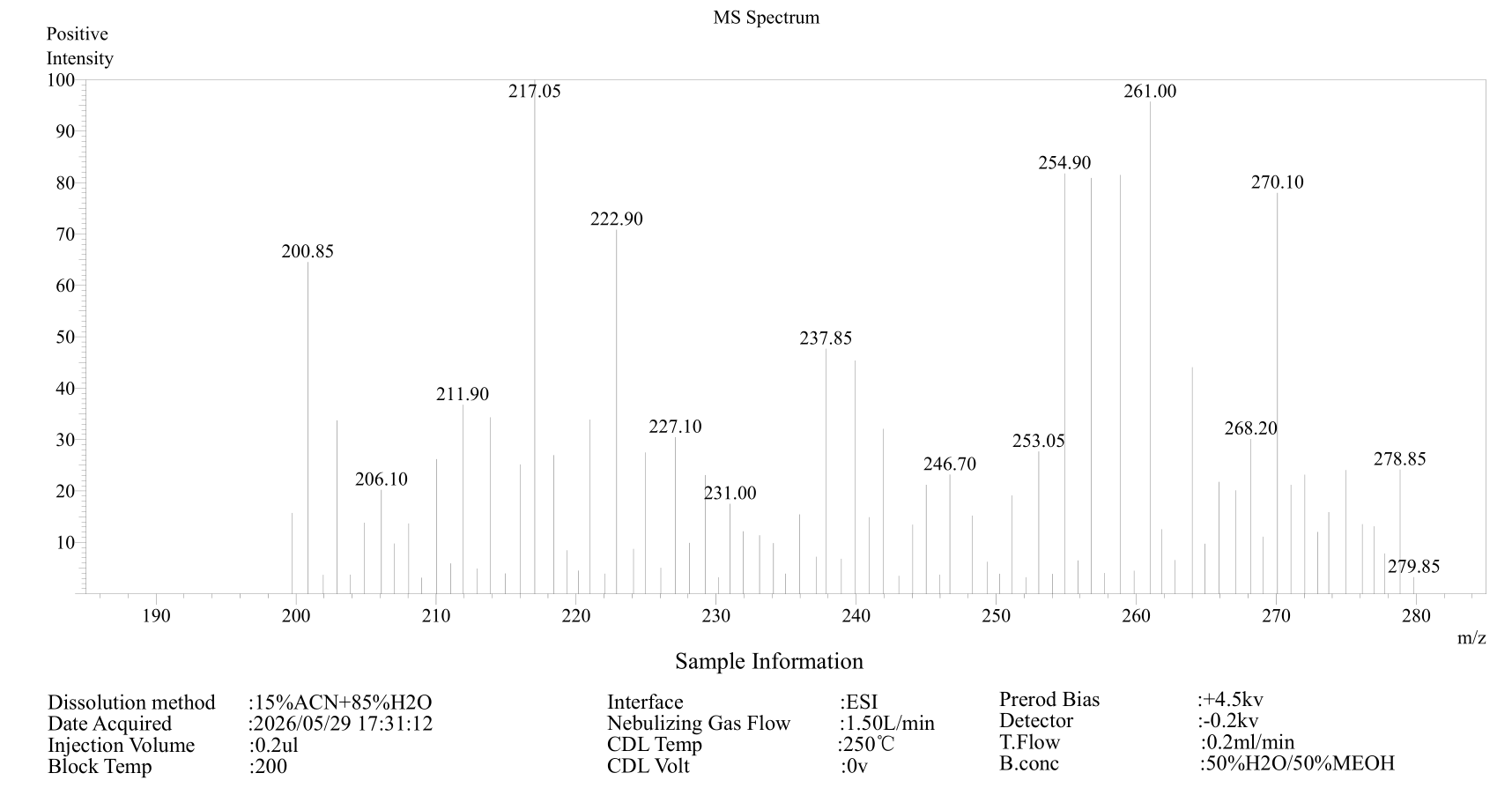


**Figure S20.** MS spectrum of the QM‑Cys adduct.


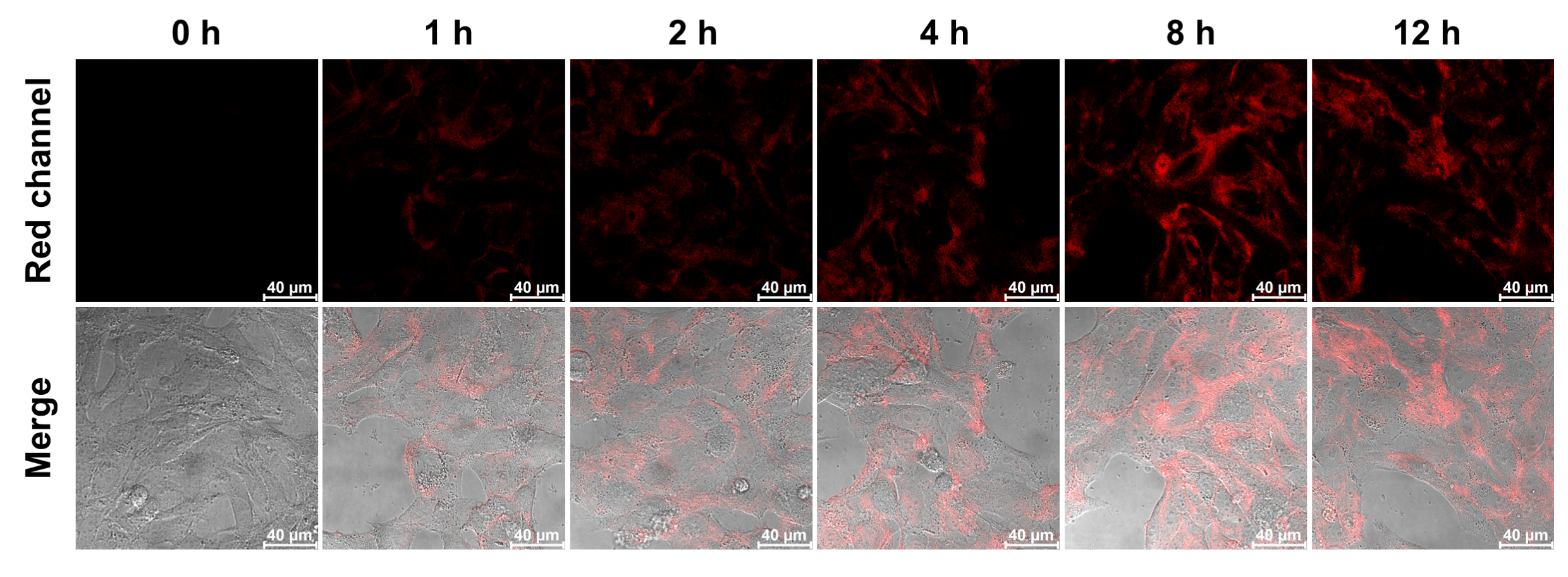


**Figure S21.** CLSM images of 143B OS cells showing cellular uptake and activation of DHUOCl-26 (10 μM) at different incubation time points.


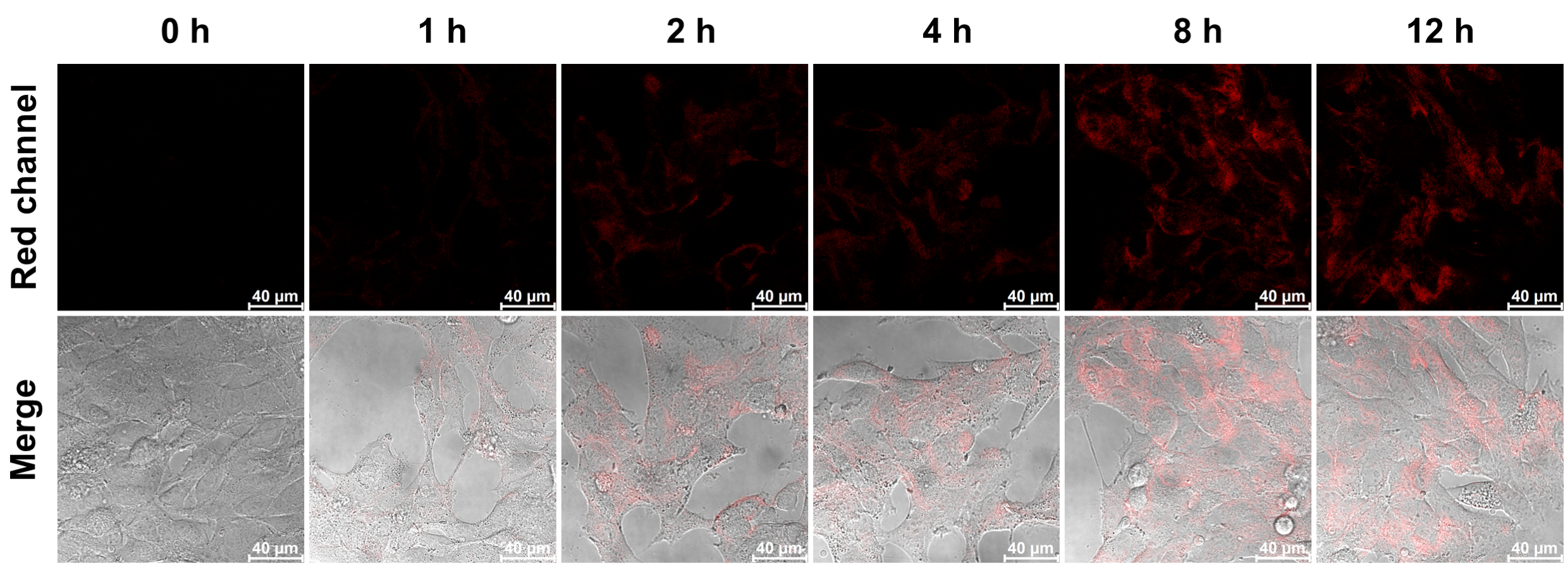


**Figure S22.** CLSM images of 143B OS cells showing cellular uptake and activation of DHUOCl-27 (10 μM) at different incubation time points.


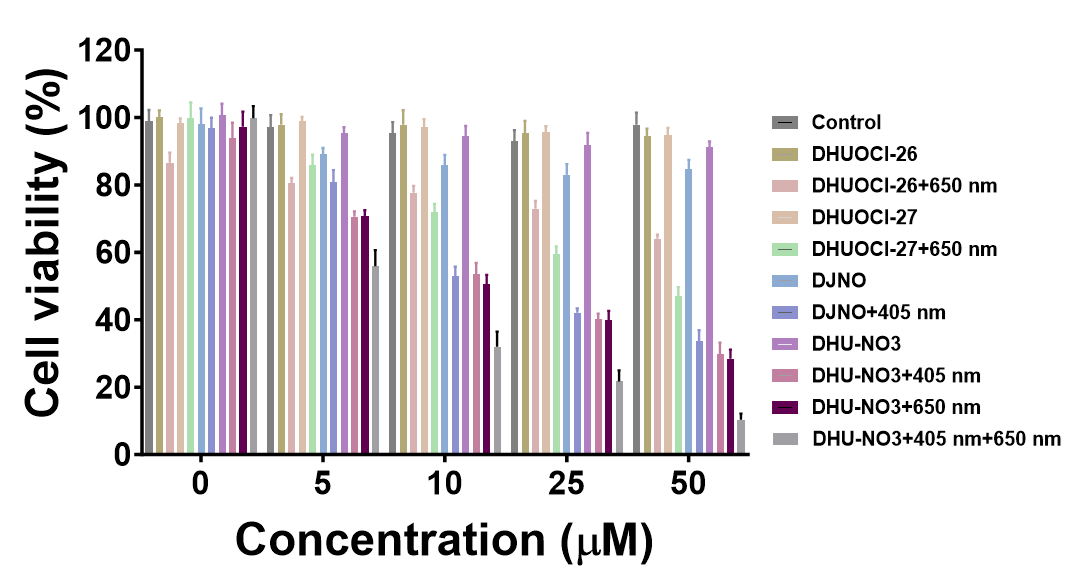


**Figure S23.** Cell viability of 143B cells treated with different concentrations of DHU-NO3 or control compounds (MB, DHUOCl-26, DHUOCl-27, DJNO) under dark conditions or following 405 nm (22 mW cm^−2^, 20 min) and 650 nm (14 mW cm^−2^, 5 min) irradiation.


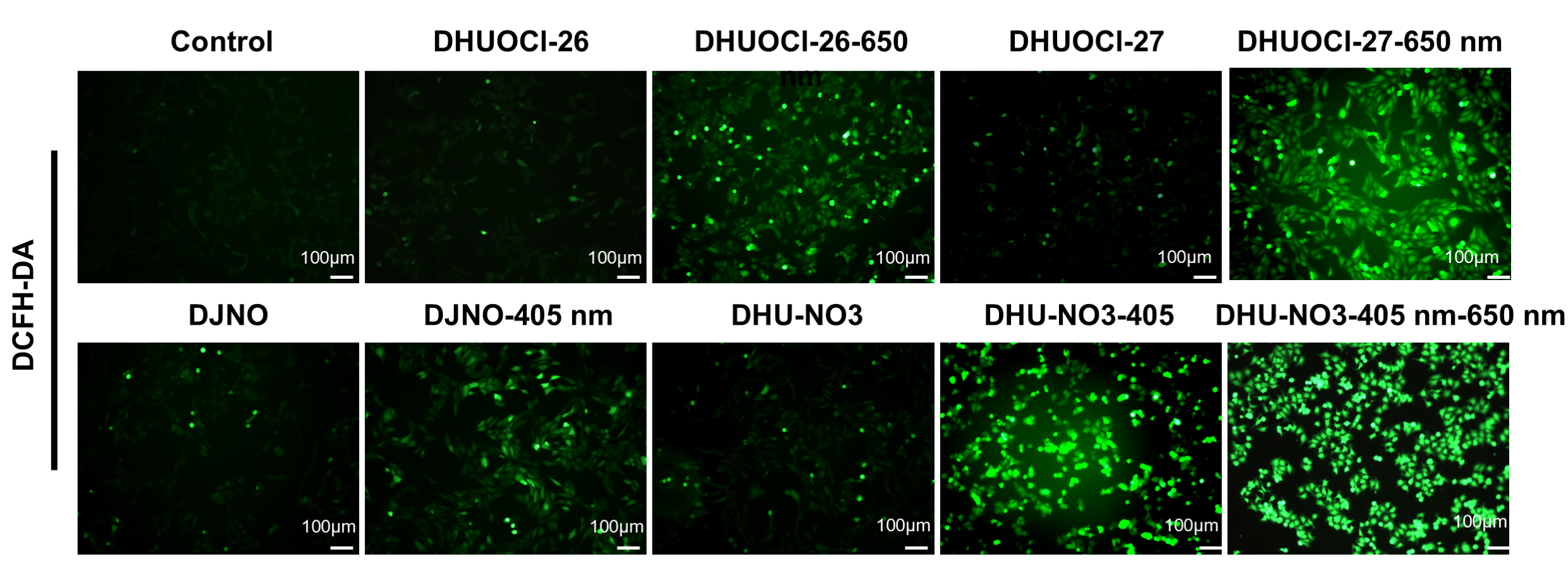


**Figure S24**. CLSM images showing intracellular levels of ROS in 143B OS cells after treatment with different compounds (5 μM) under dark conditions or following 405 nm (22 mW cm^−2^, 20 min) or 650 nm (14 mW cm^−2^, 5 min) irradiation (scale bar: 100 μm).


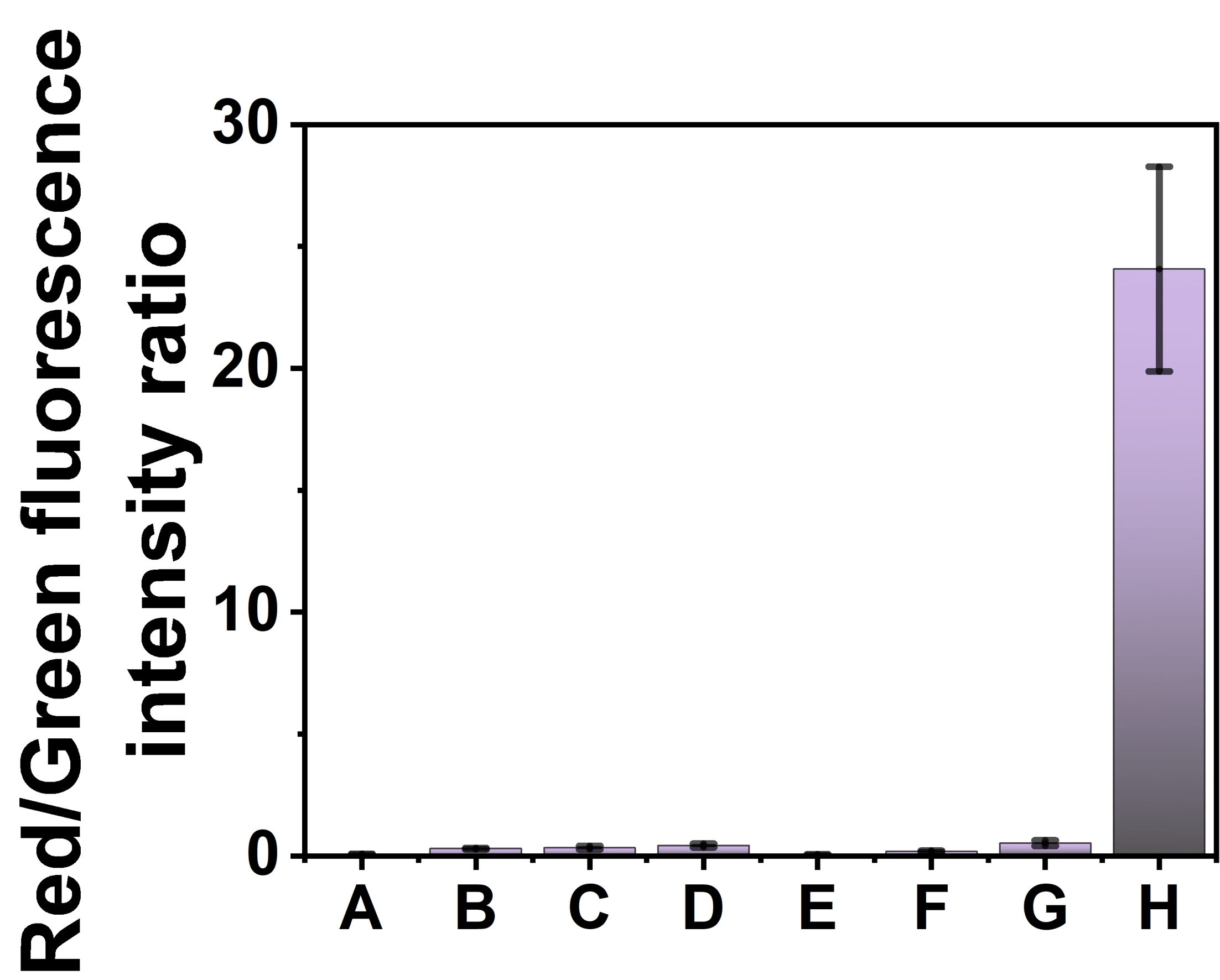


**Figure S25**. Quantitative analysis of the red/green fluorescence intensity ratio from the live/dead staining shown in Figure 4C. Cells were treated with DHU‑NO3 at the indicated concentrations (0, 10, 25, 50 μM) followed by either 405 nm irradiation alone (A-D) or combined 405/650 nm irradiation (E-H).


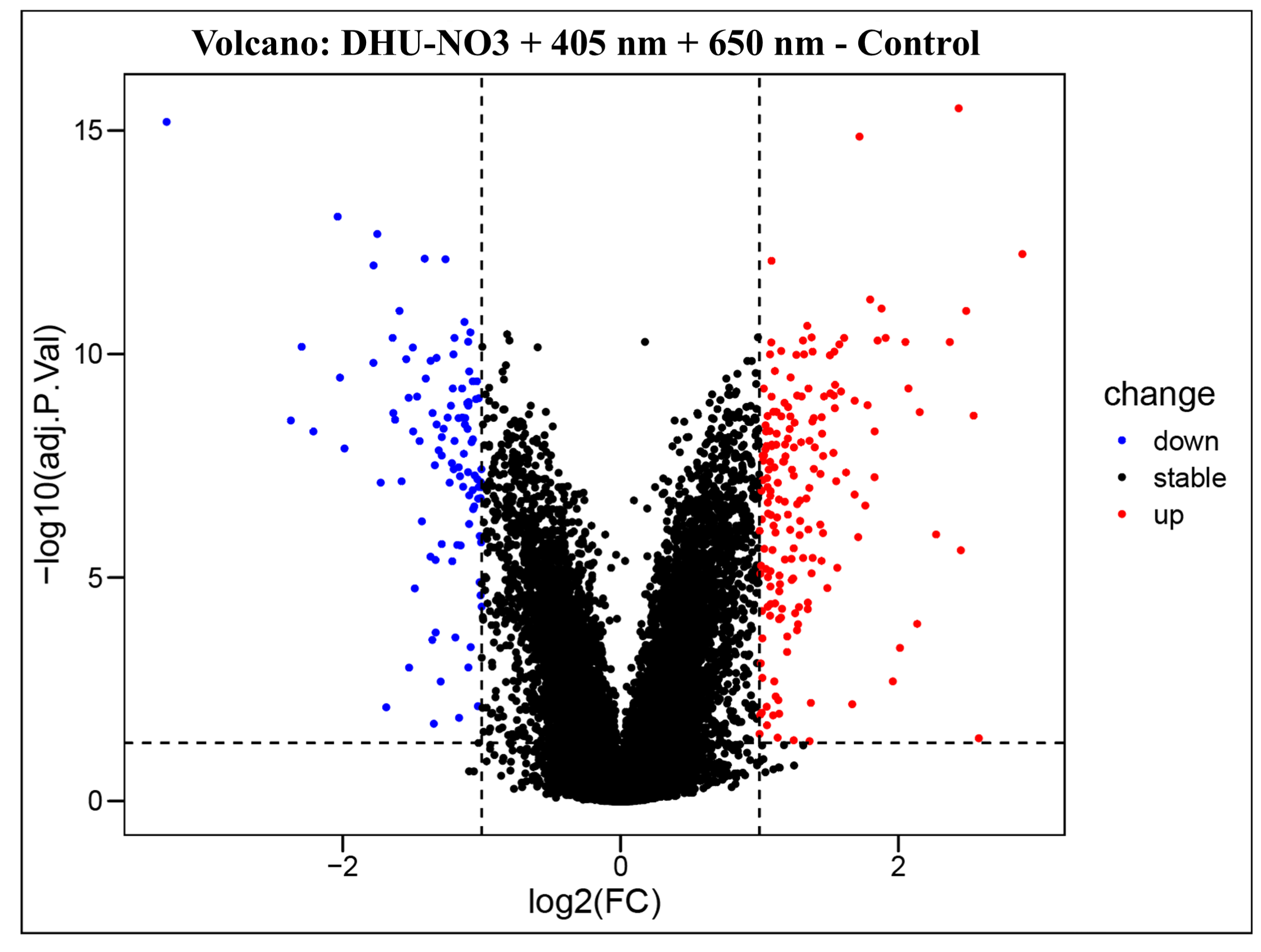


**Figure S26.** Volcano plot displaying the differentially expressed genes between the DHU-NO3 (10 μM) + 405 nm (22 mW cm^−2^, 20 min) + 650 nm (14 mW cm^−2^, 5 min) combination therapy group and the control group (n = 3).


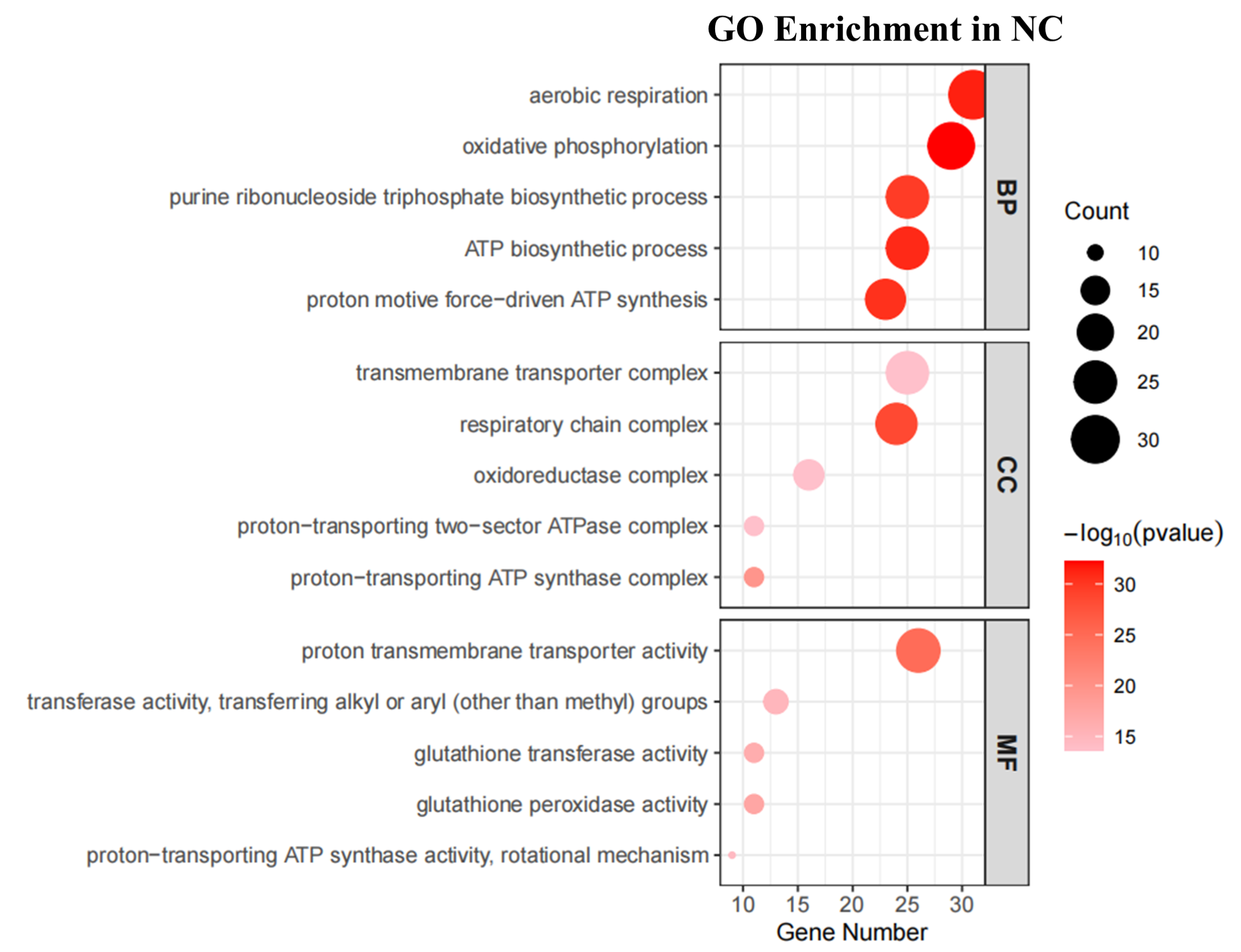


**Figure S27.** Gene Ontology (GO) enrichment analysis of genes in the control group, categorized by biological process (BP), cellular component (CC), and molecular function (MF).


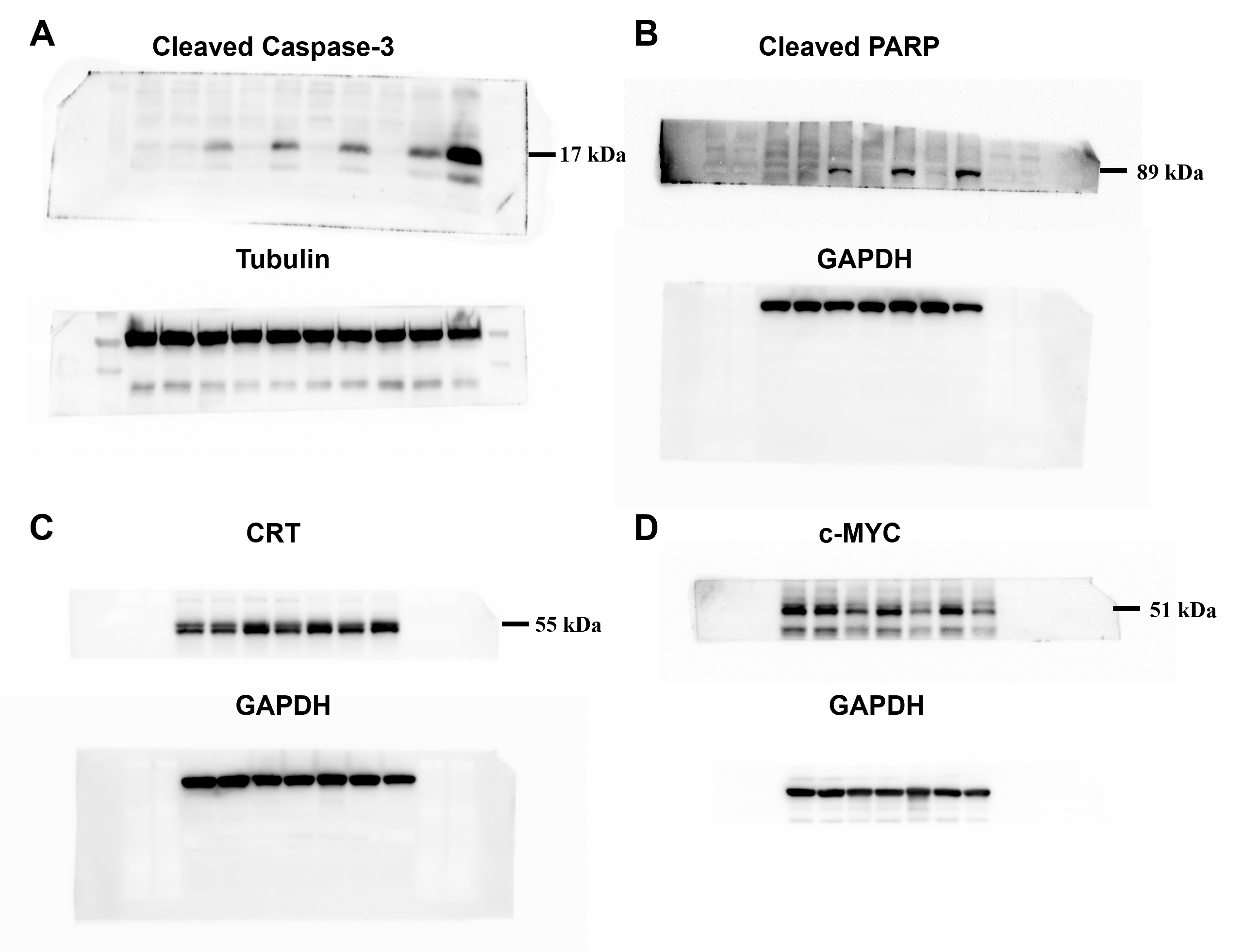


**Figure S28.** Full, uncropped Western blot membranes for (A) cleaved caspase‑3, (B) Cleaved PARP, (C) CRT, and (D) c‑MYC.


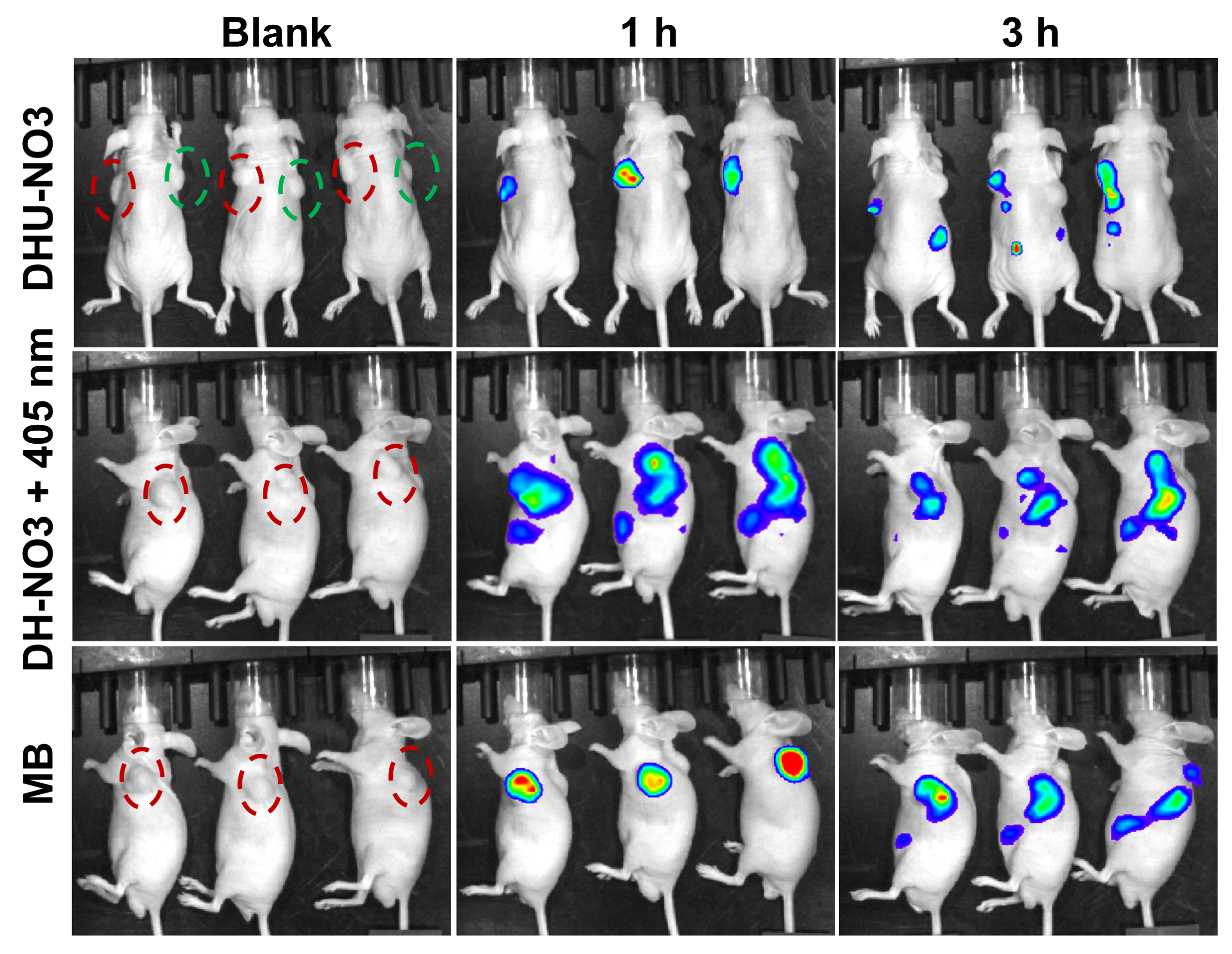


**Figure S29.** In vivo fluorescence imaging of 143B tumor-bearing mice at different time points (0, 1 h, 3 h) after intratumoral injection of DHU-NO3 (1mg/mL, 50 μL), DHU-NO3 (1mg/mL, 50 μL) followed by 405 nm irradiation, or MB (1mg/mL, 50 μL) alone.


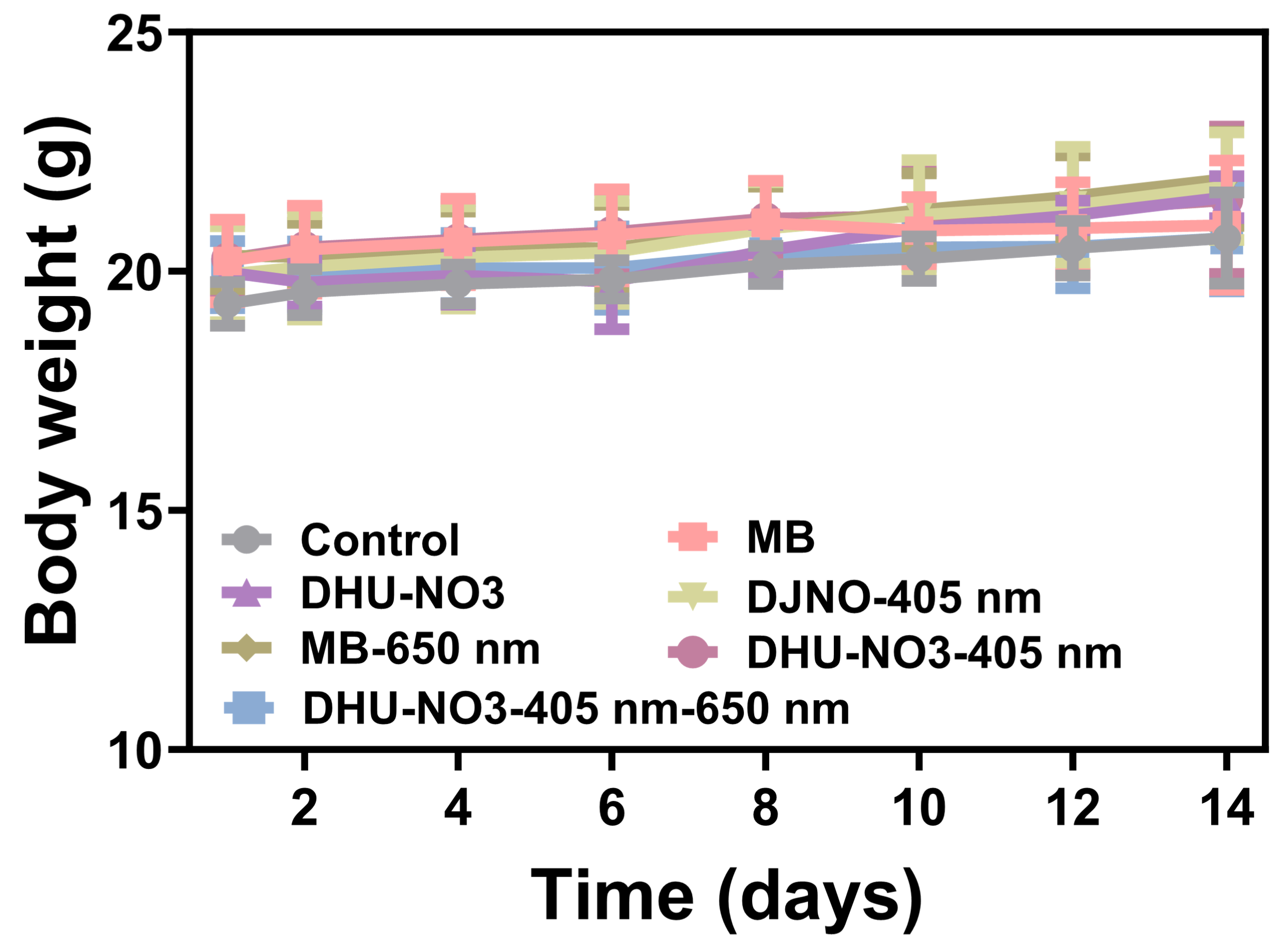


**Figure S30.** Body weight changes of mice monitored throughout the treatment course (n = 6).

**3 NMR and HRMS spectra**

**Figure S31.** ^1^H NMR spectrum of DJNO in DMSO-*d*_6_.

**Figure S32.** ^13^C NMR spectrum of DJNO in DMSO-*d*_6_.

**Figure S33.** HRMS spectrum of DJNO.

**Figure S34.** ^1^H NMR spectrum of 1-1 in DMSO-*d*_6_.

**Figure S35.** ^13^C NMR spectrum of 1-1 in DMSO-*d*_6_.

**Figure S36.** HRMS spectrum of 1-1.

**Figure S37.** ^1^H NMR spectrum of 1-2 in DMSO-*d*_6_.

**Figure S38.** ^13^C NMR spectrum of 1-2 in DMSO-*d*_6_.

**Figure S39.** HRMS spectrum of 1-2.

**Figure S40.** ^1^H NMR spectrum of DHUOCl-26 in CDCl_3_.

**Figure S41.** ^13^C NMR spectrum of DHUOCl-26 in CDCl_3_.

**Figure S42.** HRMS spectrum of DHUOCl-26.

**Figure S43.** ^1^H NMR spectrum of DHUOCl-27 in DMSO-*d*_6_.

**Figure S44.** ^13^C NMR spectrum of DHUOCl-27 in DMSO-*d*_6_.

**Figure S45.** HRMS spectrum of DHUOCl-27.

**Figure S46.** ^1^H NMR spectrum of DHU-NO3 in DMSO-*d*_6_.

**Figure S47.** ^13^C NMR spectrum of DHU-NO3 in CDCl_3_.

**Figure S48.** HRMS spectrum of DHU-NO3.

**References**

1. P. Wei, L. Liu, Y. Wen, G. Zhao, F. Xue, W. Yuan, R. Li, Y. Zhong, M. Zhang and T. Yi, *Angew. Chem. Int. Ed.*, 2019, **58**, 4547-4551.
